# The Participation of 3,3,3-Trichloro-1-nitroprop-1-ene in the [3 + 2] Cycloaddition Reaction with Selected Nitrile *N*-Oxides in the Light of the Experimental and MEDT Quantum Chemical Study

**DOI:** 10.3390/molecules26226774

**Published:** 2021-11-09

**Authors:** Karolina Zawadzińska, Mar Ríos-Gutiérrez, Karolina Kula, Przemysław Woliński, Barbara Mirosław, Tomasz Krawczyk, Radomir Jasiński

**Affiliations:** 1 Department of Organic Chemistry and Technology, Cracow University of Technology, Warszawska 24, 31-155 Cracow, Poland; karolina.zawadzinska@doktorant.pk.edu.pl (K.Z.); kkula@chemia.pk.edu.pl (K.K.); pwolinski@chemia.pk.edu.pl (P.W.); 2 Department of Organic Chemistry, University of Valencia, Dr. Moliner 50, Burjassot, 46100 Valencia, Spain; 3 Department of General and Coordination Chemistry and Crystallography, Maria Curie-Sklodowska University in Lublin, Pl. Marii Curie-Sklodowskiej 3, 20-031 Lublin, Poland; barbara.miroslaw@mail.umcs.pl; 4 Department of Chemical Organic Technology and Petrochemistry, Silesian University of Technology, Krzywoustego 4, 44-100 Gliwice, Poland; tomasz.krawczyk@polsl.pl

**Keywords:** [3 + 2] cycloaddition, nitrile oxides, nitroalkenes, reactivity, molecular mechanism, molecular electron density theory

## Abstract

The regioselective *zw-type* [3 + 2] cycloaddition (32CA) reactions of a series of aryl-substituted nitrile *N*-oxides (NOs) with trichloronitropropene (TNP) have been both experimentally and theoretically studied within the Molecular Electron Density Theory (MEDT). Zwitterionic NOs behave as moderate nucleophiles while TNP acts as a very strong electrophile in these polar 32CA reactions of forward electron density flux, which present moderate activation Gibbs free energies of 22.8–25.6 kcal·mol^−1^ and an exergonic character of 28.4 kcal·mol^−1^ that makes them irreversible and kinetically controlled. The most favorable reaction is that involving the most nucleophilic MeO-substituted NO. Despite Parr functions correctly predicting the experimental regioselectivity with the most favorable O-CCCl_3_ interaction, these reactions follow a two-stage one-step mechanism in which formation of the O-C(CCl_3_) bond takes place once the C-C(NO_2_) bond is already formed. The present MEDT concludes that the reactivity differences in the series of NOs come from their different nucleophilic activation and polar character of the reactions, rather than any mechanistic feature.

## 1. Introduction

**Scheme 1:**
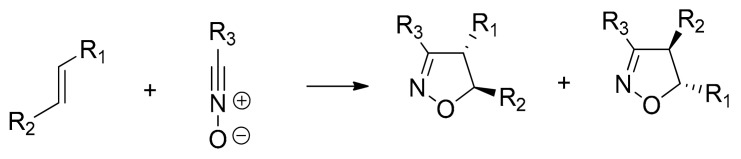
General synthesis of 2-isoxazolines via 32CA reaction of NOs with substituted alkenes.

Organic compounds containing an isoxazoline motif as a core unit are known to provide an extensive variety of biological activities such as antibacterial, antifungal, anticancer, anticonvulsant, anti-inflammatory, antiviral, and antidepressant [1,2]. One of the most successful approaches to obtain these valuable skeletons, because of its simplicity, efficiency and high selectivity, is the [3 + 2] cycloaddition (32CA) reaction of nitrile oxides (NOs), participating as the three-atom-component (TAC), with an appropriate alkenes (Scheme 1) [3,4,5].

The application of the Molecular Electron Density Theory (MEDT) to the study of 32CA reactions has allowed classifying these reactions into four different types depending on the electronic structure of TACs [6,7]. Thus, NOs, which usually present a zwitterionic electronic structure, participate in zwitterionic-type (*zw-type*) 32CA reactions, which demand favorable nucleophilic/electrophilic interactions between the reagents to take place easily experimentally. Substitution may change the parent electronic structure of the simplest TACs and, therefore, the reactivity of actual experimental TACs.

Aryl NOs are one of the most common types of experimentally used NOs. The theoretical study of the 32CA reactions of benzonitrile oxide with several alkenes such as *N*-vinyl pyrrol [8], carvone [9], tomentosin [10], 3-nitroprop-1-ene [11] and methyl acrylate [12] allowed confirming the zwitterionic reactivity of aryl NOs in *zw-type* 32CA reactions, in which, interestingly, the C-C single bond was always formed prior to the O-C one.

Trihalomethylated organic molecules also present valuable potential in pharmacy. Since the trihalomethyl group greatly withdraws electrons, its presence in compounds can hugely improve molecular properties such as bioactivity, lipophilicity and metabolic stability. Moreover, increased lipophilicity leads to an increase in therapeutic efficiency or the ability to penetrate the blood-brain barrier [13,14]. Trifluoromethylated compounds are the most commonly used molecules of this type [15], however, in recent years, trichloromethylated compounds are gaining importance as sedative-hypnotic agents [16] and antitubercular components [17], for instance.

The biological activity of these compounds can be additionally stimulated by the presence of the nitro group [18,19]. The NO_2_ group gives interesting potential possibilities for different-type functionalization in amines, nitrosocompounds, oximes, nitronates and many other [20,21,22,23]. Consequently, structures containing CX_3_ and NO_2_ functional groups are very attractive materials for further applications. The synthesis of these type of compounds is relatively easy using the corresponding 3,3,3-trihalo-1-nitroprop-1-enes as precursors. In the literature, some examples of the application of 3,3,3-trichloro-1-nitroprop-1-ene (TNP) as component of cycloaddition processes are available [24,25].

**Scheme 2:**
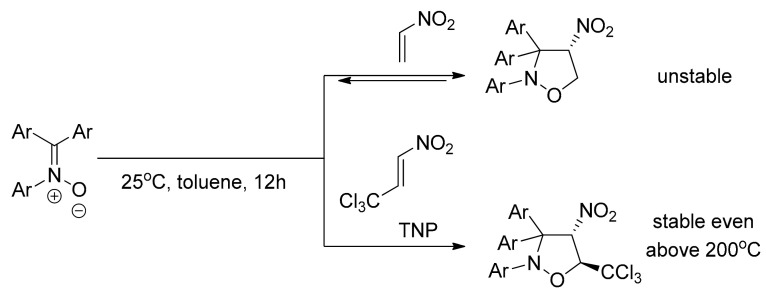
Different stability of cycloadducts obtained from 32CA reactions of triarylnitrones with different-type conjugated nitroalkenes.

The stability of cycloadducts obtained via 32CA reactions involving TNP is very variable. In some cases, the presence of the trichloromethyl CCl_3_ group strongly stabilizes the heterocyclic systems, compared to the analogs substituted with other functional groups. For example, 2,3,3-triphenyl-4-nitroisoxazolidine [26] is very unstable; it easily decomposes via retro-cycloaddition process even at 60–80 °C. However, structurally similar 2,3,3-triaryl-4-nitro-5-trichloromethylisoxazolidines melt at 106–195 °C with no decomposition (Scheme 2) [27,28].

**Scheme 3:**
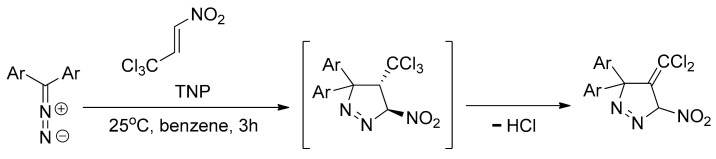
The course of the reaction between diaryldiazomethanes with TNP.

In other cases, the presence of the CCl_3_ group is not enough to stabilize the corresponding cycloadduct. For instance, diaryldiazomethanes react with TNP yielding unstable 5-nitropyrazoline systems, which surprisingly converted very fast into the corresponding 4-dimethylvinylpyrazolines via hydrochloride extrusion (Scheme 3) [24,25]. It should be underlined at this point that the HCl extrusion from molecular systems including the CCl_3_ group is very rare and was unobserved earlier in five-membered heterocycles.

**Scheme 4:**
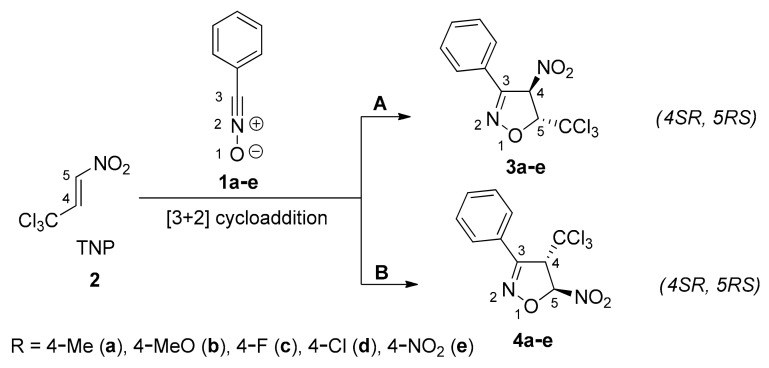
Considered 32CA reactions of NOs **1a–e** with TNP **2**.

Given the importance of the isoxazoline framework and the biological activities originated by the presence of the CCl_3_ and NO_2_ groups, we decided to shed light on the participation of TNP **2** in the 32CA reactions with a series of substituted aryl nitrile *N*-oxides **1a–e** of different nucleophilic character yielding isoxazolines **3a**–**e** (Scheme 4), as a continuation of our comprehensive study about the reactivity of this type of TAC. In particular, we performed an experimental study of the reaction course and structural analysis of reaction products, as well as theoretical investigations regarding the molecular mechanism of cycloadduct formation and nature of relevant structures along the reaction paths, in the framework of MEDT as a modern view of organic chemical reactivity [29]. Our aim is to check on the stability of isoxazolines including the CCl_3_ group and the effect of substitution on the reactivity of aryl NOs, providing significant experimental and theoretical insight to help future designs of syntheses of trichloromethyl-functionalized isoxazoline analogs with interesting potential applications.

## 2. Results and Discussion

### 2.1. Experimental Study

First, the precursors for the five aforementioned NOs **1a–e** were prepared by conversion of the corresponding aromatic aldehydes into the respective oximes via the well-known [30] reaction involving hydroxylamine hydrochloride Subsequent chlorination of the corresponding oximes to yield the hydroximinoyl chlorides precursors was carried out with *N*-chlorosuccinimide in *N*,*N*-dimethylformamide [31]. Next, the preparation of the TNP **2** was carried out via the Henry condensation between chloral hydrate and nitromethane [32]. The obtained trichloronitropropanol was converted into the expected nitroalkene via a two-step protocol consisting of esterification and acetic acid extrusion stages [33].

The considered 32CA reactions can in principle yield two regioisomeric cycloadducts (Scheme 4). The optimization of the reaction conditions was performed for the model reaction between 4-methylbenzonitrile *N*-oxide **1a** and TNP **2**. For the generation of NO **1a**, different-type protocols were tested. The relatively highest yield of the expected product was obtained (temp: 25 °C, time: 24h, molar ratio hydroxamoyl chlorides/nitroalkene 1:1,25). Regardless of the reaction conditions (Table 1), only one cycloadduct along with certain amounts of the respective furoxane (which is a secondary product of the dimerization of NOs) [34] was detected by comprehensive HPLC analysis of the reaction and post-reaction mixtures. Under optimized conditions, the yield of nitroisoxazoline **3a** is about 45%, together with the furoxan mentioned above, whose yield significantly depends on the reaction conditions (Table 1). The obtained 2-isoxazoline molecular system was isolated via simple fractional crystallization and identified based on the spectral data.

For post-analysis, HR-MS data were analyzed first. It was found that the isolated compound (**3a** or **4a**) gave a pseudo-molecular ion 320,9601mDa [M-H]^−^ (see Supplementary Materials) which corresponds with the proposed C_11_H_8_N_2_O_3_Cl_3_ molecular formula. Bands typical for the NO_2_ group [35], -N-O- [11,36], and -C=N- [11] moieties in the heterocyclic ring as well as C-Cl bond [35] vibrations were detected in its IR spectrum. Information about stereochemistry of the isolated compound was provided by ^1^H NMR spectroscopy. Protons associated with the isoxazoline ring form AX spin system. The values of J _4,5 isoxazoline ring_ coupling constants are found in the range of coupling constants typical for isoxazolines substituted at the 4- and 5- positions with *trans* configuration of the substituents.

**Figure 1:**
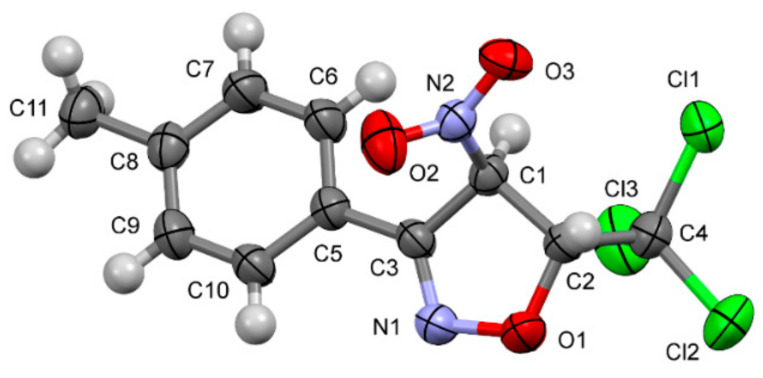
Molecular structure of NO **3a** obtained from crystal X-ray diffraction experiment (stereoisomer 4*R*,*5R*).

The single crystal X-ray diffraction experiment confirmed the formation of a heterocyclic compound with the structure presented in Figure 1. The heterocyclic 5-membered cycle is almost flat (RMSD 0.038 Å) and is rotated by 20.6(3)° relative to the aromatic ring, showing no or little electronic coupling. The torsion angle along the atoms N2-C1-C2-C4 (−128.5(2)°) shows the *ac* arrangement of the heterocyclic substituents. Weak hydrogen C-H…O/N/Cl bonds stabilize the crystal structure in a centrosymmetric triclinic space group *P*-1. No π-stacking is observed in the crystal network; instead, pnictogen, chalcogen and halogen atoms participate in short Cl…Cl, O…O/N contacts (Table S1 in Supplementary Materials). The crystal contains both enantiomers *R,R* and *S,S*.

Likewise, the other 32CA reactions involving NOs **1b**–**e** were explored. In all cases, only 3-aryl-4-nitro-5-trichloromethylisoxazolines (**3b**–**e**) were isolated and identified by spectral analyses.

### 2.2. Theoretical Study

To explain and understand the above experimental outcomes, a MEDT study of these 32CA reactions was carried out. This study is divided in the following sections: (i) analysis of the electronic structures of the reagents; (ii) analysis of the Conceptual DFT (CDFT) reactivity indicators; (iii) study of the potential energy surfaces associated with the five considered reactions; and (iv) characterization of the molecular mechanism associated with the 32CA reactions of NOs **1b** and **1e** with TNP **2**.

#### 2.2.1. Analysis of the Electronic Structures of the Reagents

**Figure 2:**
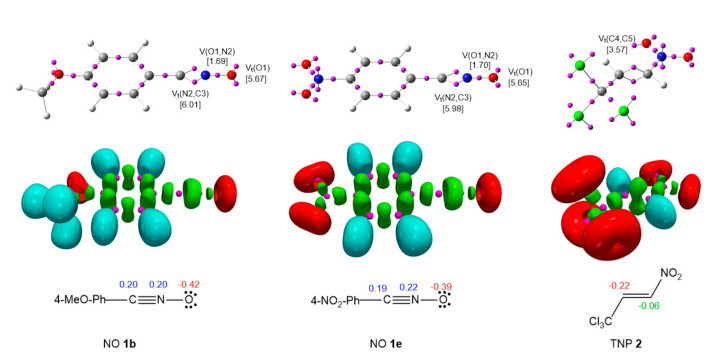
*ω*B97X-D/6-311G(d,p) ELF basin attractor positions together with some relevant valence basin populations, as well as the proposed Lewis-like structures together with the natural atomic charges, of NOs **1b,e** and TNP **2**. Populations and charges are given in average number of electrons, ē. Negative, negligible and positive charges are shown in red, green and blue colors, respectively.

One appealing procedure that provides a straightforward connection between the electron density distribution and the chemical structure is the quantum chemical analysis of Becke and Edgecombe’s Electron Localization Function (ELF) [37]. Thus, to gain insight about the reactivity of the reagents involved in the aforementioned 32CA reactions, their electronic structure was characterized through the topological analysis of the ELF and Natural Population Analysis (NPA) of their charge distribution. Herein, only the results for NOs **1b** and **1e**, and TNP **2** are commented on (Figure 2), while those for the three remaining NOs are given in Table S4 in Supplementary Material.

The topological analysis of the ELF (Figure 2) reveals a similar electronic structure for the five NOs. All of them present two V(O1) and V’(O1) monosynaptic basins integrating a total average of 5.67 ē, a V(O1,N2) disynaptic basin integrating 1.69 ē, two V(N2,C3) and V’(N2,C3) disynaptic basins integrating a total average of 6.00 ē, and a V(C3,C3′) disynaptic basin integrating an average of 2.48 ē. These features allow constructing Lewis-like structures with an O1 non-bonding region, an O1-N2 single bond, an N2-C3 triple bond and a C3-C3′ single bond. The absence of any reactive *pseudoradical* or carbenoid center and the presence of a multiple bond indicates that these NOs have a zwitterionic electronic structure just as the simplest acetonitrile oxide **5** [38] and, consequently, they will participate in *zw-type* 32CA reactions [39].

NPA shows (Figure 2) that all the NOs, regardless of the substitution, also present a similar charge distribution. In all of them, the O1 oxygen is negatively charged by ca. 0.4 e, while the N2 and C3 atoms are positively charged by ca. 0.2 e. This picture contrasts with Huisgen’s representation of NOs as 1,2-zwitterionic species [40]. On the other hand, the ethylene moiety at TNP **2** is negatively charged by −0.22 e at C4 and −0.06 e at C5, which might be unexpected taking into account the electron-withdrawing character of both CCl_3_ and NO_2_ groups.

#### 2.2.2. Analysis of the CDFT Reactivity Indices of the Reagents

CDFT [41,42] is a powerful tool to predict and understand the reactivity in organic reactions as it allows the characterization of the electrophilic and nucleophilic behaviors of molecules both qualitative and quantitatively. Thus, to discern any reactivity changes associated with the aryl substitution at NOs **1a–e** and characterize their behavior towards TNP **2**, the global CDFT reactivity indices of every reagent, namely the electronic chemical potential μ, chemical hardness η, global electrophilicity ω and global nucleophilicity *N*, were computed and analyzed (Table 2). These global indices were calculated at the B3LYP/6-31G(d) computational level to make proper classifications based on the electrophilicity and nucleophilicity scales, which were established at that level [41,42,43,44].

In polar reactions there is a flux of electron density from the species with higher electronic chemical potential [43] to the ones with lower electronic chemical potential. The electronic chemical potentials of NOs **1a–e**, which range by between −3.42 (**1b**) and −5.04 (**1e**) eV, are considerably higher than that of TNP **2**, −5.84 eV. Consequently, if the 32CA reactions between NOs **1a–e** and TNP **2** are polar (see later), the electron density will flux from the former to the latter species in a forward electron density flux (FEDF) fashion [44,45].

The chemical hardness η of the NOs range by between 4.03 (**1b**) to 5.02 (**1c**) eV; only the η of **1e** significantly differs from the rest, being a softer species. TNP **2** is the hardest species with η = 22 eV.

The electrophilicity ω indices [46] of the NOs range by between 1.19 (**1b**) and 3.15 (**1e**) eV. According to the electrophilicity ω scale [47] **1a** and **1b** are classified as moderate electrophiles with ω values below 1.5 eV, **1c** lays on the borderline between moderate and strong electrophiles, while **1d** and **1e** are strong electrophiles. Please note that **1e** is actually a very strong electrophile with a ω value above 3.0 eV. On the other hand, the nucleophilicity *N* indices of NOs range from 2.07 (**1e**) to 3.24 (**1b**) eV. Within the nucleophilicity scale [48] **1b** is classified as strong nucleophile, **1a** lays on the borderline between moderate and strong nucleophiles, while **1c–e** are classified as moderate nucleophiles.

Given that TNP **2** is classified as a very strong electrophile with ω = 3.27 eV and as a very poor nucleophile with *N* = 0.67 eV, and considering the zwitterionic nature of the NOs, it is expected that the *zw-type* 32CA reaction involving **1b** should be the most favorable one, and that the reactivity should decrease in the order **1b** > **1a** > **1c** > **1d** > **1e**. The global indices also suggest a favorable reaction between moderate-to-strong nucleophiles and a strong electrophile, in agreement with the computed activation Gibbs free energies (see later).

By approaching a non-symmetric electrophilic/nucleophilic pair along a polar or ionic process, the most favorable reactive channel is that associated with the initial two-center interaction between the most electrophilic and nucleophilic centers of the reagents [49]. Thus, to characterize the most nucleophilic and electrophilic centers of these species, the nucleophilic P_k_^−^ Parr functions of NOs **1a–e** and the electrophilic P_k_^+^ Parr functions of TNP **2** were analyzed [50]. Parr functions were calculated at the *ω*B97X-D/6-311G(d,p) level.

**Figure 3:**
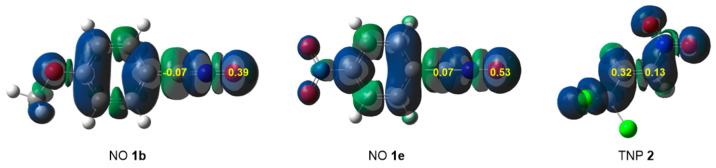
Three-dimensional representations of the Mulliken atomic spin densities of the radical cation of NOs **1b,e** and the radical anion of TNP **2**, together with the nucleophilic P_k_^−^ and the electrophilic P_k_^+^ Parr functions of NOs **1b,e** and TNP **2**, respectively.

Analysis of the nucleophilic P_k_^−^ Parr functions at the reactive sites of NOs **1a–e** clearly indicates that the most nucleophilic center is the O1 oxygen atom with values ranging from 0.39 (**1b**) to 0.53 (**1e**) (Figure 3 and Figure S1 in Supplementary Materials). Interestingly, NO **1e**, with the strong electron-withdrawing nitro NO_2_ group, has the strongest nucleophilically activated O1 oxygen, while NO **1b**, with the strong electron-donating methoxy MeO group, has the weakest, yet strong, activation. The C3 carbon is not nucleophilically activated in any case. On the other hand, the C4 carbon adjacent to the trichloromethyl group at TNP **2** is more than twice as electrophilically activated, P_k_^+^ = 0.32, as the C5 carbon near the NO_2_ group, P_k_^+^ = 0.13. Consequently, the most favorable reaction path along a polar 32CA reaction between NOs **1a–e** and TNP **2** would correspond to the O1-C4 interaction, which agrees with the experimental regioselectivity (Scheme 4). In addition, the null nucleophilic activation of the C3 carbon of these NOs accounts for the full regioselectivity experimentally found.

#### 2.2.3. Study of the Potential Energy Surfaces Associated with the 32CA Reactions between NOs **1a–e** and TNP 2

**Scheme 5:**
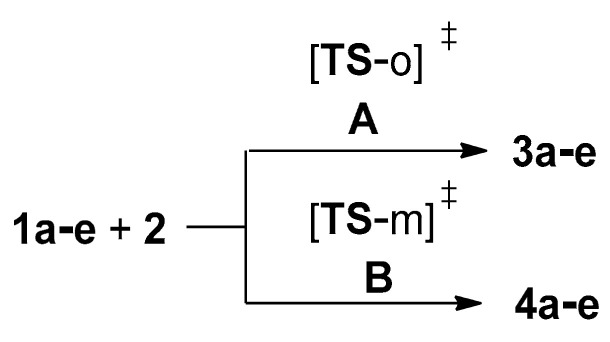
Regioisomeric reactions paths associated with the 32CA reactions of NOs **1a–e** with TNP **2**. ‡: the symbol indicates a transition state.

**Figure 4:**
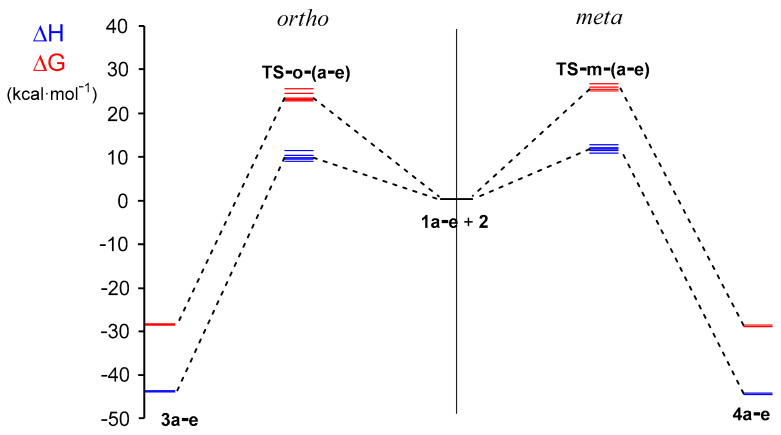
*ω*B97X-D/6-311G (d,p) enthalpy, in blue, and Gibbs free energy, in red, profiles, in kcal·mol^−1^, for the competitive reaction paths associated with the 32CA reactions of NOs **1a–e** with TNP **2**.

Due to the non-symmetry of the reagents and the linear structure of NOs **1a–e**, the 32CA reactions between NOs **1a–e** and TNP **2** can only take place along two regioisomeric reaction paths depending on the relative position of the R-aryl substituent of NOs **1a–e** with respect to the nitro NO_2_ group of TNP **2**; namely *A*, associated with the O1-C4 two-center interaction leading to *ortho* isoxazolines **3a–e**, and *B*, associated with the C3-C4 two-center interaction leading to *meta* isoxazolines **4a–e** (Scheme 5). Analysis of the potential energy surfaces allowed finding only one TS for each reaction path, indicating that these reactions follow a one-step mechanism. Electronic energies in gas phase and in solvent are given in Table S6 in Supplementary Materials, full thermodynamic data are gathered in Table S7 in Supplementary Materials, while relative enthalpies and Gibbs free energies are graphically represented in Figure 4.

The activation enthalpies of the considered reaction paths range by between 8.4 (**TS-o-b**) and 12.6 (**TS-m-e**) kcal·mol^−1^, while the reaction enthalpies are found in the narrow range of −43.8 and −44.6 kcal·mol^−1^. After inclusion of room temperature and entropies to enthalpies, the relative Gibbs free energies are by between 13.6 and 15.8 kcal·mol^−1^ higher as a consequence of the unfavorable entropies associated with these bimolecular processes. Thus, the activation Gibbs free energies reach values of 22.8–26.8 kcal·mol^−1^, while formation of cycloadducts is exergonic by 28.3–28.9 kcal·mol^−1^.

Some appealing conclusions can be drawn from these energy profiles: (i) in agreement with the experimental outcomes, the five 32CA reactions are *ortho* regioselective, as the *meta* TSs are found between 1.2 and 2.3 kcal·mol^−1^ above the *ortho* ones in the Gibbs free energy profiles (Figure 4); (ii) the activation Gibbs free energies decrease in the order NO_2_ > Cl > F > Me > MeO, in complete agreement with the analysis of the CDFT indices (see Section 2.2.2); (iii) these 32CA reactions are kinetically and thermodynamically favorable, and their strong exergonic character makes them irreversible, formation of the corresponding cycloadducts being therefore under kinetic control; (iv) the strong exergonic character of the reactions points out the very high stability of cycloadducts presenting the trichloromethyl CCl_3_ group. Please note that the corresponding analog of **3b** without CCl_3_ is 1.8 kcal·mol^−1^ less stable; and finally, (v) the substitution at benzonitrile *N*-oxide has an important role increasing the reaction rates. Please note that the activation Gibbs free energies of the reaction of unsubstituted benzonitrile *N*-oxide with TNP **2** is 23.5 kcal·mol^−1^, which means a reaction rate 3.5 times lower.

Taking into account that these 32CA reactions take place through favorable kinetic control, the Eyring-Polanyi equation [51] was used to estimate the composition of the reaction mixture.

(1) $$k=\frac{kk_{B}T}{h}e^{-\frac{\Delta G^{‡}}{RT}}$$

From this equation, the relative reaction rate constants *k_rel_* can be obtained as:

(2) $$k_{rel}=e^{-\frac{\Delta \Delta G^{‡}}{RT}}$$

were $\Delta \Delta G^{‡}$ is the difference between the relative activation Gibbs free energies of two TSs, R the gas ideal constant, and T the reaction temperature.

The expected percentage of *ortho* and *meta* isoxazolines for each reaction are reported in Table 3. Considering the *ω*B97X-D/6-311G(d,p) Gibbs free energies in tetrahydrofuran (THF) and the reaction temperature, the *ortho* and *meta* isoxazolines should be obtained in an average 94.7:5.3 relation; while the reactions of **1a–c** present a similar regioselectivity, those involving **1d,e** are slightly less regioselective. This result indicates that while the *ortho* isoxazolines **3a–e** are expected to be the major isomers, formation of the *meta* isoxazolines **4a–e** should be negligible, in clear agreement with the experimental outcomes (Scheme 4). This result emphasizes that the extent of regioselectivity cannot be predicted simply by ΔΔ*G*^‡^ between regioisomeric TSs.

**Figure 5:**
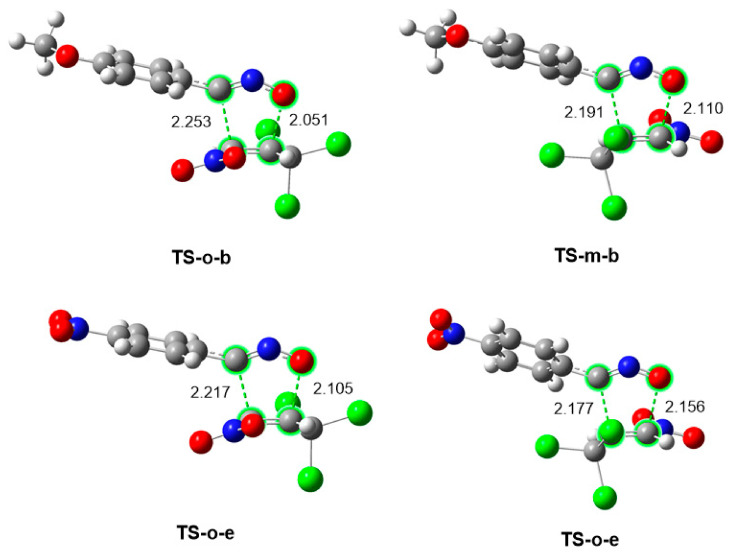
*ω*B97X-D/6-311G(d,p) optimized geometries in THF of the regioisomeric TSs involved in the 32CA reaction of NOs **1b** with TNP **2**.

The optimized geometries of the regioisomeric TSs involved in the 32CA reactions of NO **1b** with TNP **2** in THF are displayed in Figure 5, while the rest are given in Figure S1 in Supplementary Materials. The distances do not significantly change with the substitution of the TAC. Thus, at the more favorable *ortho* TSs, the average O1-C4 distance is 2.074 Å, while the average C3-C5 distance is 2.234 Å. At the *meta* TSs, the average distance between the O1 and C5 atoms is 2.133 Å, while that between the C3 and C4 atoms is 2.184 Å. Some information can be extracted from these geometrical data: (i) the more favorable *ortho* TSs are geometrically more asymmetric, Δd = 0.155 Å, than the *meta* ones, Δd = 0.051 Å, which can be considered almost symmetrical; (ii) the O-C distance is always shorter than the C-C one; (iii) taking into account the C-C and C-O bond formation distances characterized from previous ELF topological analyses [6], it can be concluded that neither O-C nor C-C bond formation has started yet at any of these TSs; and (iv) the heterocyclic 5-membered cycle in NO **1b** is rotated by 11.7° relative to the aromatic ring and the torsion angle along the atoms N2-C1-C2-C4 is −117.3°, which are 8.9° and 11.2° lower than the respective experimental X-ray diffraction data (see Section 2.1).

The polar character of these *zw-type* 32CA reactions was evaluated by computing the global electron density transfer (GEDT) [52,53] at the TSs. The GEDT values in THF range by between 0.11 and 0.15 e. These values indicate that the corresponding *zw-type* 32CA reactions have somewhat polar character. The *ortho* TS are only a hundredth more polar than the *meta* ones. The GEDT increases in the order NO_2_ > Cl > F > Me > MeO, in agreement with the increased nucleophilic character in the same order (see Section 2.2.2). This trend in the polar character of the reactions follows the same trend as the computed activation energies, emphasizing the relation between the polarity and reactivity in *zw-type* 32CA reactions; [39] i.e., the stronger the polar character, the more favorable the reaction. Consequently, the different nucleophilic character of NOs **1a–e** is responsible for their different reactivity.

#### 2.2.4. Characterization of the Bond Formation Process

The Bonding Evolution Theory (BET) [54], which combines the topological analysis of the ELF with catastrophes theory [54], allows characterizing the electron density rearrangement along a reaction path and, thus, enables a description of the bonding changes associated with a given molecular mechanism [55,56].

The molecular mechanism of *zw-type* 32CA reactions involving NOs and different types of ethylene derivatives has already been recently studied [2,9,10,12,53,57,58,59,60,61]. As a general outlook, these reactions start with the depopulation of the C≡N triple bond of the NO framework and the C=C double bond of the ethylene derivative, which gives rise to the creation of the corresponding carbon *pseudoradical* centers [62]. The electron density at the oxygen atom of the NO also experiences some rearrangement. Then, the bond formation takes place either by coupling of two carbon *pseudoradical* centers in the case of the C-C single bond [52], or by donation of the oxygen non-bonding electron density to the remaining carbon *pseudoradical* center in the case of the C-O single bond. In polar reactions, the formation of the first single bond takes place between the most nucleophilic center of the nucleophile and the most electrophilic center of the electrophile as characterized by CDFT and the Parr functions. Accordingly, in *zw-type* 32CA reactions of NOs, this should be the C-O single bond (see Section 2.2.2); however, previous BET studies [57,63] have shown that regardless of the polar character, the C-C single bond forms first in 32CA reactions of NOs.

**Figure 6:**
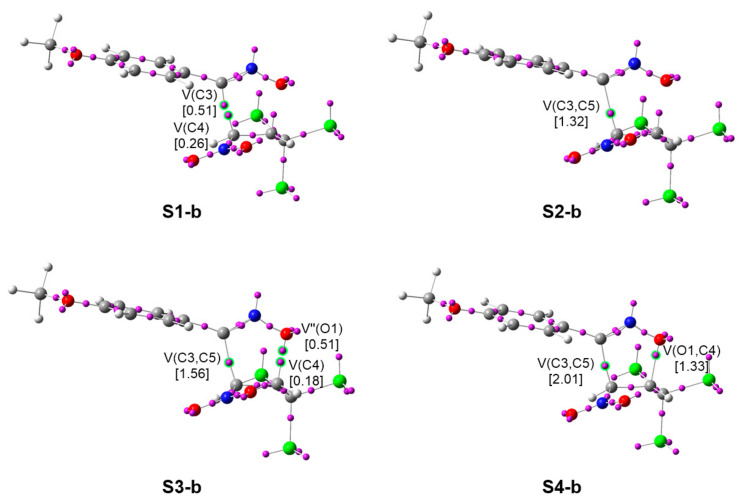
Attractor positions of the ELF basins of the selected structures involved in the O1-C4 and C3-C5 single bond formation along the regioisomeric path *A* of the *zw-type* 32CA reaction between NO **1b** and TNP **2**. Electron populations, in average number of electrons, ē, are given in brackets.

Thus, the characterization of the bond formation processes in the 32CA reactions of NOs **1a–e** with TNP **2** would not only add valuable information about this question, but would also help identifying any remarkable change in the molecular mechanisms which may account for the observed reactivity differences. To do so, BET was applied over the 32CA reactions of NOs **1b,e** with TNP **2** in THF. Only the structures involved in the bond formation are reported and discussed herein. ELF attractor positions for the selected structures involved only in the most favorable reaction of NO **1b** are displayed in Figure 6.

Analysis of the bonding changes along the 32CA reactions of NOs **1b,e** with TNP **2** shows very similar bond formation processes, regardless of the substitution of the TAC. This behavior is in agreement with the similar electronic structures of these NOs (see Section 2.2.1). At the two TSs, both the N2-C3 and C4-C5 bonding regions have been depopulated with respect to the separated reagents (see Section 2.2.1) to 4.29 (**TS-o-b**) and 4.40 (**TS-o-e**) ē, which can already be considered a double bond and an overpopulated single bond, respectively, but no *pseudoradical* center is observed yet. Further approach of the two molecular frameworks leads to the creation of *pseudoradical* centers at the three carbon atoms involved. At **S1**, the two C3 and C5 *pseudoradical* centers reach populations of ca. 0.5 and 0.8 ē, just before merging each other at **S2** to give rise to the formation of the first C3-C5 single bond, at a C-C distance of ca. 1.97 Å, with an electron population of 1.48 (**S2-b**) and 1.33 (**S2-e**) ē. At **S3**, right before the formation of the second O1-C4 single bond, the non-bonding electron densities associated with both the O1 oxygen and the C4 carbon reach ca. 5.74 and 0.19 ē. At **S4**, formation of the O1-C4 single bond takes place at an O-C distance of ca. 1.76 Å by donation of ca. 0.6 ē of the O1 oxygen electron density to the C4 *pseudoradical* center, whose population has slightly decreased to ca. 0.2 ē. The new O1-C4 bond has a population of ca. 0.8 ē. At the final isoxazolines **3b,e**, the O1-C4 and C3-C5 bonds reach ca. 1.34 and 2.02 ē. As formation of the second O1-C4 single bond occurs when the first C3-C5 formed bond has already reached 78% (**1b**) and 83% (**1e**) of its final population at **3b,e**, it can be considered that these polar *zw-type* 32CA reactions follow a *two-stage one-step* mechanism [64]. Indeed, the evolution of the forming bond distances is considerably synchronous (Table 4 and Table 5), thus emphasizing that geometrical analyses are not valid to characterize the stage of the bond formation progress.

The most relevant finding from this BET study is that despite the Parr functions accounting for the solely isolation of regioisomeric isoxazolines **3b,e**, formation of the C3-C5 single bond involving the least nucleophilic and electrophilic centers of the reagents takes place before the expected more favorable O1-C5 one. This unexpected pattern is also observed in previous BET studies of polar *zw-type* 32CA reactions of NOs [57,63]. On the other hand, the similar bonding changes taking place along the 32CA reactions of the two most electronically different NOs **1b** and **1e** with TNP **2** indicates that the computed reactivity changes among the series of NOs **1a–e** are mainly due to their different nucleophilic character and polarity of the corresponding reactions, rather than any structural or mechanistic reason.

## 3. Methods and Procedures

### 3.1. Experimental

#### 3.1.1. Analytical Techniques

HPLC analyses was done using a Knauer device with a UV-VIS detector (LiChrospher 18-RP 10 µm column, eluent: 80% methanol). M.p. values were measured on the Boetius apparatus and were uncorrected. IR spectra were derived from the FTS Nicolet IS 10 spectrophotometer. UV/Vis spectra were recorded using spectrometer UV-5100 BIOSENS and was determined for the 200–500nm range. NMR spectra were registered on an Agilent 400 MHz spectrometer using CDCl_3_ as a solvent. HR-MS spectra were acquired on a UPLC-MS-MS Waters Xevo G2 QTof instrument with ESI ionization.

#### 3.1.2. RTG Analysis

The diffraction intensities were collected at room temperature with the use of SuperNova X-ray diffractometer with Atlas S2 CCD detector using the mirror-monochromatized CuK*α* radiation (*λ* = 1.54184 Å). The phase problem was solved by direct methods using the SHELXS-97 program and the structure model refined by the full-matrix least-squares method on F^2^ using the SHELXL-97 program implemented in Olex2.refine package [65,66]. Details on crystal structure refinement and geometry are given in Tables S1 in Supplementary Materials.

CCDC No. 2,115,222 contains the supplementary crystallographic data for this paper. These data can be obtained free of charge via http://www.ccdc.cam.ac.uk/conts/retrieving.html (accessed on 20 August 2021) (or from the Cambridge Crystallographic Data Centre, 12, Union Road, Cambridge CB2 1EZ, UK; fax: +44-1223-336033).

#### 3.1.3. Materials

The components of the cycloaddition were prepared according to procedures described in the literature. In particular, nitrile *N*-oxides were generated in situ from respective hydroxamoyl chlorides, obtained via chlorination of respective oximes according to known procedures [31]. The 3,3,3-trichloro-1-nitroprop-1-ene was synthetized via three-step protocol starting from nitromethane and chloral hydrate [32]. Commercially available (Sigma Aldrich, St. Louis, MO, USA) chemicals were used as solvents and as components for the preparation of addends.

##### Cycloaddition between Nitrile *N*-Oxides and 3,3,3-Trichloro-1-nitroprop-1-ene—General Procedure

Erlenmeyer flask containing 10 cm^3^ of THF was placed in an ice bath. Next, 0.003 mole of 3,3,3-trichloro-1-nitroprop-1-ene and 0.0024 mole of hydroximinoyl chloride was added and stirred for 10 min. Then 0.0015 mole of K_2_CO_3_ was dosed in small portions during 30 min period. After that time, the ice bath was removed. Change of mixture color and turbidity was observed. The mixture was left for 24 h with constant stirring. The solvent was evaporated and remaining solid was mixed with diethyl ether and filtrated to remove insoluble side products. The ether was removed under vacuum, and remaining crude product was washed with light petroleum ether and crystallized from ethanol. A white crystalline solid was obtained.

### 3.2. Computational

DFT calculations were performed using the hybrid ωB97X-D functional [67] which includes long-range exchange (denoted by X) correction as well as the semiclassical London-dispersion correction (indicated by suffix-D). The standard 6-311G(d,p) [68] basis set was used, which includes d-type polarization for second row elements and p-type polarization functions for hydrogen atoms. The Berny method was used in optimizations [69,70]. A similar theory level has been commonly used for the mechanistic research aspects of cycloaddition reactions [71,72,73,74,75] The TSs were characterized through frequency analysis, presenting only one imaginary frequency. The intrinsic reaction coordinate (IRC) paths [76] were computed to find the unique connection between the TSs and the minimum stationary points using the second order González-Schlegel integration method [77,78]. Solvent effects of THF were considered by full optimization of the gas-phase structures at the same computational level using the polarizable continuum model (PCM) [79,80] in the framework of the self-consistent reaction field (SCRF) [81,82,83]. Values of ωB97X-D/6-311G(d,p) enthalpies, entropies and Gibbs free energies in THF were calculated with standard statistical thermodynamics at 298.15 K and 1 atm [68].

The GEDT [52] values were estimated by a natural population analysis (NPA) [84,85] using the equation GEDT(f) = $\Sigma_{q\in f}q$, were q are the atoms of a framework (f) at the TSs. CDFT reactivity indices [41,42] were calculated at the B3LYP/6-31G(d) computational level because the electrophilicity and nucleophilicity scales were established at that level. The global electrophilicity ω index [46] is given by the following expression, $\omega =\frac{\mu^{2}}{2\eta }$, in terms of the electronic chemical potential μ and chemical hardness η. Both quantities may be approached in terms of the one-electron energies of the frontier molecular orbitals HOMO and LUMO, *ε_H_* and *ε_L_*, as $\mu =\frac{\left(\varepsilon_{H}+\varepsilon_{L}\right)}{2}$ and $\eta =\varepsilon_{L}-\varepsilon_{H}$, respectively [41]. The global nucleophilicity *N* index, ref. [86] based on the HOMO energies obtained within the Kohn-Sham scheme, ref. [87] is defined as *N* = ε_H_(Nu) − ε_H_(TCE), where tetracyanoethylene (TCE) is the reference. Parr functions were calculated at the *ω*B97X-D/6-311G(d,p) level. All computations were carried out with the Gaussian 16 suite of programs [88].

The topology of the ELF [37] of the *ω*B97X-D/6-311G(d,p) monodeterminantal wavefunctions was carried out using the TopMod [89] package with a cubical grid of step size of 0.1 Bohr. The analysis of the bond formation processes was performed by applying the Bonding Evolution Theory (BET) along the corresponding reaction paths; 312 (methoxy) and 337 (nitro) nuclear configurations along the IRC paths were considered. GaussView program [90] was used to visualize molecular geometries of all the systems as well as the position of the ELF basin attractors.

## 4. Conclusions

The *zw-type* 32CA reactions of a series of aryl-substituted NOs **1a–e** with TNP **2** have been both experimentally and theoretically studied within the framework of MEDT. These reactions, carried out at room temperature, during 24 h and using THF as solvent, lead to the formation of single regioisomeric isoxazolines **3a–e** with moderate to high yields. Regioisomeric isoxazolines **4a–e** were not detected and only little amounts of furoxan, coming from the dimerization of the NOs, were isolated.

The topological analysis of the ELF of NOs **1a–e** allows characterizing their zwitterionic structure, just as that of the simplest acetonitrile oxide, thus being able to participate in *zw-type* 32CA reactions. No relevant change is observed in the electronic structure within this series of TACs upon substitution, suggesting that the substitution would not lead to major changes in reactivity.

Thus, CDFT shows that NOs **1a–e** behave as moderate nucleophiles while TNP **2** acts as a very strong electrophile in 32CA reactions of FEDF. According to the nucleophilic character of NOs **1a–e**, it is expected that the *zw-type* 32CA reaction involving **1b** should be the most favorable one, and that the reactivity should decrease in the order **1b** > **1a** > **1c** > **1d** > **1e**. The nucleophilic and electrophilic Parr functions characterize the O1 oxygen of the NOs as their most nucleophilic center and the C4 carbon of TNP **2** as its most electrophilic center. Therefore, the expected more favorable regioisomeric isoxazolines are **3a–e**, in agreement with the experimental outcomes.

The activation Gibbs free energies of the five 32CA reactions are very similar, ranging from 22.8 to 25.6 kcal·mol^−1^, while formation of isoxazolines **3a–e** is exergonic by ca. 28.4 kcal·mol^−1^, thus being irreversible and kinetically controlled. All of them present an *ortho* regioselectivity, in agreement with the experimental obtaining of only **3a–e**. The values of GEDT computed at the TSs confirm the polar character of these *zw-type* 32CA reactions.

The BET study of the bond formation processes along the 32CA reactions involving NOs **1b,e** indicates a similar bonding pattern for both reactions. Consequently, the reactivity differences in the series of NOs **1a–e** come from the different nucleophilic activation of the TACs and polar character of the reactions, rather than any mechanistic feature. Interestingly, despite the more favorable two-center interaction between the O1 and C4 atoms, formation of the C3-C5 bond takes place first, through a *two-stage one-step* mechanism in which formation of the second O1-C4 bond takes place once the C3-C5 bond is practically formed by up to 80%.

## Figures and Tables

**Table 1:** Optimization of reaction conditions for model 32CA reaction between TNP **2** and nitrile *N*-oxide **1a**.

Method	Base	Temp. [°C]	Time [h]	Molar Ratio Hydroxamoyl Chlorides/Nitroalkene	Yield [%]	
					Isoxazoline	Furoxan
1	K_2_CO_3_	25 (r.t.)	24	1:1.25	40.0	1.0
		66 (reflux)	6	1:1.25	40.0	8.1
2	TEA	25 (r.t.)	24	1:1.25	44.4	0.9
		66 (reflux)	6	1:1.25	43.5	5.0
1	K_2_CO_3_	25 (r.t.)	24	1:2	9.8	0.8
		66 (reflux)	6	1:2	25.6	7.5
2	TEA	25 (r.t.)	24	1:2	20.8	0.7
		66 (reflux)	6	1:2	47.6	10.6

**Table 2:** B3LYP/6-31G(d) electronic chemical potential μ, chemical hardness η, electrophilicity ω and nucleophilicity *N* indices, in eV, of NOs **1a–e** and TNP **2**.

Compound	μ	η	ω	*N*
**1b**	−3.42	4.91	1.19	3.24
**1a**	−3.69	4.97	1.37	2.95
**1c**	−3.85	5.02	1.47	2.76
**1d**	−4.12	4.96	1.71	2.52
**1e**	−5.04	4.03	3.15	2.07
**2**	−5.84	5.22	3.27	0.67

**Table 3:** Calculated composition of the reaction mixture for the 32CA reactions of NOs **1a–e** with TNP **2**.

	3	4
Cl (**d**)	91.4	8.6
MeO (**b**)	98.0	2.0
NO2 (**e**)	88.7	11.3
F (**c**)	97.1	2.9
Me (**a**)	98.1	1.9

**Table 4:** Electron populations of the most relevant ELF valence basins and O1-C4 and C3-C5 distances for **TS-o-b**, isoxazoline **3b** and the structures **S1-b**–**S4-b** involved in the formation of the O1-C4 and C4-C5 single bonds along the more favorable regioisomeric path *A* of the 32CA reaction between NO **1b** and TNP **2**. Populations are given in average number of electrons, ē, and distances in angstroms, Å.

	TS-o-b	S1-b	S2-b	S3-b	S4-b	3b
d(O1-C4)	2.051	1.865	1.856	1.760	1.751	1.428
d(C3-C5)	2.253	1.999	1.988	1.862	1.850	1.512
V(O1,N2)	1.40	1.31	1.30	1.23	1.23	1.07
V(N2)	2.09	2.44	2.46	2.58	2.60	2.80
V(N2,C3)	2.17	1.81	1.80	1.75	1.74	1.67
V′(N2,C3)	2.12	1.90	1.89	1.81	1.81	1.60
V(C4,C5)	3.45	2.47	2.45	2.29	2.27	2.03
V(O1)	2.83	2.73	2.72	2.65	2.62	2.47
V′(O1)	2.82	2.89	2.89	2.59	2.56	2.42
V′′(O1)				0.51		
V(C4)		0.26	0.27	0.18		
V(C3)		0.51				
V(C5)		0.79				
V(C3,C5)			1.32	1.56	1.57	2.01
V(O1,C4)					0.79	1.33

**Table 5:** Electron populations of the most relevant ELF valence basins and O1-C4 and C3-C5 distances for **TS-o-e**, isoxazoline **3e** and the structures **S1-e**–**S4-e** involved in the formation of the O1-C4 and C4-C5 single bonds along the more favorable regioisomeric path *A* of the 32CA reaction between NO **1e** and TNP **2**. Populations are given in average number of electrons, ē, and distances in angstroms, Å.

	TS-o-e	S1-e	S2-e	S3-e	S4-e	3e
d(O1-C4)	2.105	1.932	1.924	1.770	1.761	1.429
d(C3-C4)	2.217	1.972	1.960	1.771	1.761	1.509
V(O1,N2)	1.44	1.34	1.33	1.23	1.23	1.08
V(N2)	2.01	2.39	2.41	2.61	2.62	2.79
V(N2,C3)	2.25	1.83	1.83	1.74	1.73	1.67
V′(N2,C3)	2.15	1.91	1.90	1.78	1.78	1.61
V(C4,C5)	3.43	2.49	2.47	2.23	2.21	2.04
V(O1)	2.82	2.74	2.73	2.64	2.62	2.47
V′(O1)	2.79	2.84	2.85	2.57	2.54	2.40
V′′(O1)				0.53		
V(C4)		0.23	0.24	0.20		
V(C3)		0.56				
V(C5)		0.75				
V(C3,C5)			1.33	1.67	1.68	2.02
V(O1,C4)					0.81	1.34

## Data Availability

The data presented in this study are available on request from the corresponding author.

## Supplementary Materials

The following are available online. Table S1: Crystal X-ray diffraction data for Δ^2^-isoxazoline (**3a**); Table S2: Bond lengths (Å) and valence angles (°) for Δ^2^-isoxazoline (**3a**); Table S3: Geometry of interactions in of crystal Δ^2^-isoxazoline (**3a**); Table S4: Populations of the most relevant ELF valence basins of NOs **1a,c,d**, in average number of electrons, e; Table S5: B3LYP/6-31G(d) HOMO and LUMO energies used for the calculation of the global CDFT indices of NOs **1a–e**, in eV; Table S6: Nucleophilic P_k_^−^ Parr functions at the O1 and C3 atoms of NOs **1a,c,d**; Table S7: *ω*B97X-D/6-311G(d,p) electronic energies in gas phase and in tetrahydrofuran (THF), in a.u., of the reagents and stationary points involved in the 32CA reactions of NOs **1a–e** with TNP **2**; Table S8: *ω*B97X-D/6-311G(d,p) enthalpies (H, in a.u.), entropies (S, in cal·mol^−1^K^−1^), and Gibbs free energies (G, in a.u.), and the relative ones with respect to the separated reagents, computed at 25 °C, in THF, of the reagents and stationary points involved in the 32CA reaction of NOs **1a–e** with TNP **2**; Figure S1: *ω*B97X-D/6-311G(d,p) optimized geometries, in THF, of the regioisomeric TSs involved in the 32CA reaction of NOs **1a,c,d** with TNP **2**; Figure S2: *ω*B97X-D/6-311G(d,p) intrinsic reaction coordinate paths, in THF, of the 32CA reactions between NOs **1a,e** and TNP **2**.

## Sample Availability

Samples of the compounds are not available from the authors.

## References

[1] Prajapti S.K., Shrivastava S., Bihade U., Gupta A.K., Naidu V.G.M., Banerjee U.C., Babu B.N. (2015). Synthesis and biological evaluation of novel Δ^2^-isoxazoline fused cyclopentane derivatives as potential antimicrobial and anticancer agents. MedChemComm.

[2] Domingo L.R., Emamian S., Salami M., Ríos-Gutiérrez M. (2016). Understanding the molecular mechanism of [3 + 2] cycloaddition reaction of benzonitrile oxide toward an *N*-vinylpyrrole derivative with the aid of ELF topological analysis. J. Phys. Org. Chem..

[3] Ren J., Wang S., Ni H., Yao R., Liao C., Ruan B. (2015). Synthesis, Characterization and Antitumor Activity of Novel Ferrocene-Based Amides Bearing Pyrazolyl Moiety. J. Inorg. Organomet. Polym..

[4] Houk K.N., Sims J., Watts C.R., Luskus L.J. (1973). Origin of reactivity, regioselectivity, and periselectivity in 1,3-dipolar cycloadditions. J. Am. Chem. Soc..

[5] Rai N.S., Kalluraya B., Lingappa B., Shenoy S., Puranic V.G. (2008). Convenient access to 1,3,4-trisubstituted pyrazoles carrying 5-nitrothiophene moiety via 1,3-dipolar cycloaddition of sydnones with acetylenic ketones and their antimicrobial evaluation. Eur. J. Med. Chem..

[6] Ríos-Gutiérrez M., Domingo L.R. (2019). Unravelling the Mysteries of the [3 + 2] Cycloaddition Reactions. Eur. J. Org. Chem..

[7] Ríos-Gutiérrez M., Domingo L.R., Ghodsi F. (2021). Unveiling the Different Reactivity of Bent and Linear Three-Atom-Components Participating in [3 + 2] Cycloaddition Reactions. Organics.

[8] Ríos-Gutiérrez M., Chafaa F., Khorief Nacereddine A., Djerourou A., Domingo L.R. (2016). A DFT study of [3 + 2] cycloaddition reactions of an azomethine imine with *N*-vinyl pyrrole and *N*-vinyl tetrahydroindole. J. Mol. Graph. Model..

[9] Ríos-Gutiérrez M., Domingo L.R., Esseffar M., Oubella A., Ait Itto M.Y. (2020). Unveiling the Different Chemical Reactivity of Diphenyl Nitrilimine and Phenyl Nitrile Oxide in [3 + 2] Cycloaddition Reactions with (R)-Carvone through the Molecular Electron Density Theory. Molecules.

[10] Zeroual A., Ríos-Gutiérrez M., El Idrissi M., El Alaoui El Abdallaoui H., Domingo L.R. (2019). An MEDT study of the mechanism and selectivities of the [3 + 2] cycloaddition reaction of tomentosin with benzonitrile oxide. Int. J. Quantum Chem..

[11] Mirosław B., Babyuk D., Łapczuk-Krygier A., Kącka-Zych A., Demchuk O.M., Jasiński R. (2018). Regiospecific formation of the nitromethyl-substituted 3-phenyl-4,5-dihydroisoxazole via [3 + 2] cycloaddition. Monatsh. Chem..

[12] Ndassa I.M., Adjieufack A.I., Mbadcam Ketcha J., Berski S., Ríos-Gutiérrez M., Domingo L.R. (2017). Understanding the reactivity and regioselectivity of [3 + 2] cycloaddition reactions between substituted nitrile oxides and methyl acrylate. A molecular electron density theory study. Int. J. Quantum Chem..

[13] Yavari I., Malekafzali A., Eivazzadeh-Keihan R., Skoulika S., Alivaisi R. (2016). A one-pot synthesis of trichloromethylated pyrimidines from trichloroacetimidamides and acetylenic esters. Tetrahedron Lett..

[14] Furuya T., Kamlet A.S., Ritter T. (2011). Catalysis for fluorination and trifluoromethylation. Nature.

[15] Purser S., Moore P.R., Swallow S., Gouverneur V. (2008). Fluorine in medicinal chemistry. Chem. Soc. Rev..

[16] Darko L.L. (1971). Synthesis and biological properties of certain trichloromethyl compounds. J. Med. Chem..

[17] Srivastav M.K., Shantakumar S.M. (2013). Design and Synthesis of novel 2-trichloromethyl-4-substituted quinazoline derivatives as anti-tubercular agents. Chem. Sci. Trans..

[18] Boguszewska-Czubara A., Kula K., Wnorowski A., Biernasiuk A., Popiolek Ł., Miodowski D., Demchuk O.M., Jasiński R. (2019). Novel functionalized β-nitrostyrenes: Promising candidates for new antibacterial drugs. Saudi Pharm. J..

[19] Boguszewska-Czubara A., Łapczuk-Krygier A., Rykała K., Biernasiuk A., Wnorowski A., Popiolek Ł., Maziarka A., Hordyjewska A., Jasiński R. (2016). Novel synthesis scheme and in vitro antimicrobial evaluation of a panel of (E)-2-aryl-1-cyano-1-nitroethenes. J. Enzym. Inhib. Med. Chem..

[20] Ono N. (2001). Conversion of Nitro Compounds into Other Compounds. The Nitro Group in Organic Synthesis.

[21] Ballini R., Petrini M. (2004). Recent synthetic developments in the nitro to carbonyl conversion (Nef reaction). Tetrahedron.

[22] Sibi M.P., Manyem S. (2000). Enantioselective Conjugate Additions. Tetrahedron.

[23] Halimehjani A.Z., Namboothini I.N.N., Hooshmand S.E. (2014). Nitroalkenes in the synthesis of carbocyclic compounds. RSC Adv..

[24] Kula K., Dobosz J., Jasiński R., Kącka-Zych A., Łapczuk-Krygier A., Mirosław B., Demchuk O.M. (2020). [3 + 2] Cycloaddition of diaryldiazomethanes with (E)-3,3,3-trichloro-1-nitroprop-1-ene: An experimental, theoretical and structural study. J. Mol. Struct..

[25] Kula K., Kącka-Zych A., Łapczuk-Krygier A., Wzorek Z., Nowak A., Jasiński R. (2021). Experimental and theoretical mechanistic study on the thermal decomposition of 3,3-diphenyl-4-(trichloromethyl)-5-nitropyrazoline. Molecules.

[26] Jasiński R. (2009). The question of the regiodirection of the [2 + 3] cycloaddition reaction of triphenylnitrone to nitroethene. Chem. Heterocycl. Compd..

[27] Jasiński R., Mróz K., Kącka-Zych A. (2016). Experimental and theoretical DFT study on synthesis of sterically crowded 2,3,3,(4)5- tetrasubstituted-4-nitroisoxazolidines via 1,3-dipolar cycloaddition reactions between ketonitrones and conjugated nitroalkenes. J. Heterocycl. Chem..

[28] Jasiński R., Mróz K. (2015). Kinetic aspects of [3 + 2] cycloaddition reactions between (E)-3,3,3-trichloro-1-nitroprop-1-ene and ketonitrones. React. Kinet. Mech. Catal..

[29] Domingo L.R. (2016). Molecular Electron Density Theory: A modern view of reactivity in organic chemistry. Molecules.

[30] Kula K., Dresler E., Demchuk O.M., Jasiński R. (2015). New aldimine *N*-oxides as precursors for preparation of heterocycles with potential biological activity. Przem. Chem..

[31] Liu K.-C., Shelton B.R., Howe R.K. (1980). A particularly convenient preparation of benzohydroximinoyl chlorides (nitrile oxide precursors). J. Org. Chem..

[32] Perekalin W., Lipina E.S., Berestovitskaya V.M., Efremov D.A. (1994). The Nitro-Aldol (Henry) Reaction. Nitroalkenes: Conjugated Nitrocompounds.

[33] Kącka A.B., Jasiński R.A. (2016). A density functional theory mechanistic study of thermal decomposition reactions of nitroethyl carboxylates: Undermine of “pericyclic” insight. Heteroat. Chem..

[34] Barbaro G., Battaglia A., Dondoni A. (1970). Kinetics and mechanism of dimerisation of benzonitrile *N*-oxides to furazan *N*-oxides. J. Chem. Soc. B.

[35] Silverstein R.M., Webster F.X., Kiemle D.J., Bryce D.L. (2014). Spectrometric Identification of Organic Compounds.

[36] Fryźlewicz A., Łapczuk-Krygier A., Kula K., Demchuk O.M., Dresler E., Jasiński R. (2020). Regio- and stereoselective synthesis of nitro-functionalized analogs of nicotine. Chem. Heterocycl. Compd..

[37] Becke A.D., Edgecombe K.E. (1990). A simple measure of electron localization in atomic and molecular systems. J. Chem. Phys..

[38] Adjieufack A.I., Ndassa I.M., Ketcha Mbadcam J., Ríos-Gutiérrez M., Domingo L.R. (2017). Steric interactions controlling the syn diastereofacial selectivity in the [3 + 2] cycloaddition reaction between acetonitrile oxide and 7-oxanorborn-5-en-2-ones: A molecular electron density theory study. J. Phys. Org. Chem..

[39] Domingo L.R., Aurell M.J., Pérez P. (2014). A DFT analysis of the participation of zwitterionic TACs in polar [3 + 2] cycloaddition reactions. Tetrahedron.

[40] Huisgen R. (1961). 1,3-dipolar cycloaddition. Proc. Chem. Soc..

[41] Parr R.G., Yang W. (1989). Density Functional Theory of Atoms and Molecules.

[42] Domingo L.R., Ríos-Gutiérrez M., Pérez P. (2016). Applications of the Conceptual Density Functional Theory Indices to Organic Chemistry Reactivity. Molecules.

[43] Parr R.G., Pearson R.G. (1983). Absolute Hardness: Companion Parameter to Absolute Electronegativity. J. Am. Chem. Soc..

[44] Domingo L.R., Ríos-Gutiérrez M., Pérez P. (2020). A molecular electron density theory study of the participation of tetrazines in aza-Diels–Alder reactions. RSC Adv..

[45] Domingo L.R., Kula K., Ríos-Gutiérrez M. (2020). Unveiling the reactivity of cyclic azomethine ylides in [3 + 2] cycloaddition reactions within the Molecular Electron Density Theory. Eur. J. Org. Chem..

[46] Parr R.G., Szentpaly L.V., Liu S. (1999). Electrophilicity Index. J. Am. Chem. Soc..

[47] Domingo L.R., Aurell M.J., Pérez P., Contreras R. (2002). Quantitative characterization of the global electrophilicity power of common diene/dienophile pairs in Diels–Alder reactions. Tetrahedron.

[48] Jaramillo P., Domingo L.R., Chamorro E., Pérez P. (2008). A further exploration of a nucleophilicity index based on the gas-phase ionization potentials. J. Mol. Struct. THEOCHEM.

[49] Aurell M.J., Domingo L.R., Pérez P., Contreras R. (2004). A theoretical study on the regioselectivity of 1,3-dipolar cycloadditions using DFT-based reactivity indexes. Tetrahedron.

[50] Domingo L.R., Pérez P., Sáez J.A. (2013). Understanding the local reactivity in polar organic reactions through electrophilic and nucleophilic Parr functions. RSC Adv..

[51] Evans M.G., Polanyi M. (1935). Some applications of the transition state method to the calculation of reaction velocities, especially in solution. Trans. Faraday Soc..

[52] Domingo L.R. (2014). A new C–C bond formation model based on the quantum chemical topology of electron density. RSC Adv..

[53] Zawadzińska K., Kula K. (2021). Application of β-phosphorylated nitroethenes in [3 + 2] cycloaddition reactions involving benzonitrile *N*-oxide in the light of DFT computational study. Organics.

[54] Krokidis X., Noury S., Silvi B. (1997). Characterization of elementary chemical processes by catastrophe theory. J. Phys. Chem. A.

[55] Berski S., Andrés J., Silvi B., Domingo L.R. (2003). The Joint Use of Catastrophe Theory and Electron Localization Function to Characterize Molecular Mechanisms. A Density Functional Study of the Diels−Alder Reaction between Ethylene and 1,3-Butadiene. J. Phys. Chem. A.

[56] Polo V., Andrés J., Berski S., Domingo L.R., Silvi B. (2008). Understanding reaction mechanisms in organic chemistry from catastrophe theory applied to the electron localization function topology. J. Phys. Chem. A.

[57] Domingo L.R., Picher M.T., Arroyo P., Sáez J.A. (2006). 1,3-Dipolar Cycloadditions of Electrophilically Activated Benzonitrile *N*-Oxides. Polar Cycloaddition versus Oxime Formation. J. Org. Chem..

[58] Kula K., Zawadzińska K. (2021). Local nucleophile-electrophile interactions in [3 + 2] cycloaddition reactions between benzonitrile *N*-oxide and selected conjugated nitroalkenes in the light of MEDT computational study. Curr. Chem. Lett..

[59] Jasiński R., Jasińska E., Dresler E. (2017). A DFT computational study of the molecular mechanism of [3 + 2] cycloaddition reactions between nitroethene and benzonitrile *N*-oxides. J. Mol. Model..

[60] Łapczuk-Krygier A., Kącka-Zych A., Kula K. (2019). Recent progress in the field of cycloaddition reactions involving conjugated nitroalkenes. Curr. Chem. Lett..

[61] Jasinski R., Dresler E. (2020). On the Question of Zwitterionic Intermediates in the [3 + 2] Cycloaddition Reactions: A Critical Review. Organics.

[62] Domingo L.R., Sáez J.A. (2011). Understanding the Electronic Reorganization along the Nonpolar [3 + 2] Cycloaddition Reactions of Carbonyl Ylides. J. Org. Chem..

[63] Domingo L.R., Acharjee N. (2021). Unveiling the Chemo- and Regioselectivity of the [3 + 2] Cycloaddition Reaction between 4-Chlorobenzonitrile Oxide and β-Aminocinnamonitrile with a MEDT Perspective. ChemistrySelect.

[64] Domingo L.R., Sáez J.A., Zaragozá R.J., Arnó M. (2008). Understanding the Participation of Quadricyclane as Nucleophile in Polar [2σ + 2σ + 2π] Cycloadditions toward Electrophilic π Molecules. J. Org. Chem..

[65] Sheldrick G.M. (2008). A short history of SHELX. Acta Crystallogr. Sect. A.

[66] Dolomanov O.V., Bourhis L.J., Gildea R.J., Howard J.A.K., Puschmann H. (2009). OLEX2: A complete structure solution, refinement and analysis program. J. Appl. Crystallogr..

[67] Chai J.-D., Head-Gordon M. (2008). Long-range corrected hybrid density functionals with damped atom-atom dispersion corrections. Phys. Chem. Chem. Phys..

[68] Hehre W.J., Radom L., Schleyer P.v.R., Pople J.A. (1986). Ab Initio Molecular Orbital Theory.

[69] Schlegel H.B. (1982). Optimization of equilibrium geometries and transition structures. J. Comput. Chem..

[70] Schlegel H.B., Yarkony D.R. (1994). Geometry Optimization on Potential Energy Surfaces. Modern Electronic Structure Theory.

[71] Kula K., Łapczuk-Krygier A. (2018). A DFT computational study on the [3 + 2] cycloaddition between parent thionitrone and nitroethene. Curr. Chem. Lett..

[72] Mlostoń G., Jasiński R., Kula K., Heimgartner H. (2020). A DFT Study on the Barton-Kellogg Reaction—The molecular mechanism of the formation of thiiranes in the reaction between diphenyldiazomethane and diaryl thioketones. Eur. J. Org. Chem..

[73] Jasiński R. (2021). On the Question of Stepwise [4 + 2] Cycloaddition Reactions and Their Stereochemical Aspects. Symmetry.

[74] Domingo L.R., Kula K., Ríos-Gutiérrez M., Jasiński R. (2021). Understanding the Participation of Fluorinated Azomethine Ylides in Carbenoid-Type [3 + 2] Cycloaddition Reactions with Ynal Systems: A Molecular Electron Density Theory Study. J. Org. Chem..

[75] Jasiński R. (2018). β-Trifluoromethylated nitroethenes in Diels-Alder reaction with cyclopentadiene: A DFT computational study. J. Fluor. Chem..

[76] Fukui K. (1970). Formulation of the reaction coordinate. J. Phys. Chem..

[77] Gonzalez C., Schlegel H.B. (1990). Reaction path following in mass-weighted internal coordinates. J. Phys. Chem..

[78] Gonzalez C., Schlegel H.B. (1991). Improved algorithms for reaction path following: Higher order implicit algorithms. J. Chem. Phys..

[79] Tomasi J., Persico M. (1994). Molecular Interactions in Solution: An Overview of Methods Based on Continuous Distributions of the Solvent. Chem. Rev..

[80] Simkin Y., Sheikhet I. (1995). Quantum Chemical and Statistical Theory of Solutions: A Computational Approach.

[81] Cossi M., Barone V., Cammi R., Tomasi J. (1996). Ab initio study of solvated molecules: A new implementation of the polarizable continuum model. Chem. Phys. Chem..

[82] Cances E. (1997). A new integral equation formalism for the polarizable continuum model: Theoretical background and applications to isotropic and anisotropic dielectrics. J. Chem. Phys..

[83] Barone V., Cossi M., Tomasi J. (1998). Geometry optimization of molecular structures in solution by the polarizable continuum model. J. Comput. Chem..

[84] Reed A.E., Weinstock R.B., Weinhold F. (1985). Natural population analysis. J. Chem. Phys..

[85] Reed A.E., Curtiss L.A., Weinhold F. (1988). Intermolecular interactions from a natural bond orbital, donor-acceptor viewpoint. Chem. Rev..

[86] Domingo L.R., Chamorro E., Pérez P. (2008). Understanding the Reactivity of Captodative Ethylenes in Polar Cycloaddition Reactions. A Theoretical Study. J. Org. Chem..

[87] Kohn W., Sham L.J. (1965). Self-Consistent Equations Including Exchange and Correlation Effects. Phys. Rev..

[88] Frisch M.J., Trucks G.W., Schlegel H.B., Scuseria G.E., Robb M.A., Cheeseman J.R., Scalmani G., Barone V., Petersson G.A., Fox D.J. (2016). Gaussian 16.

[89] Noury S., Krokidis X., Fuster F., Silvi B. (1999). Computational tools for the electron localization function topological analysis. Comput. Chem..

[90] Dennington R., Keith T.A., Millam J.M. (2016). Gauss View.

