# Supplementary material for: The Participation of 3,3,3-Trichloro-1-nitroprop-1-ene in the [3 + 2] Cycloaddition Reaction with Selected Nitrile N-Oxides in the Light of the Experimental and MEDT Quantum Chemical Study
Source: Molecules. 2021 Nov 9;26(22):6774. doi: 10.3390/molecules26226774 (PMC8622200; doi:10.3390/molecules26226774)
Supplement: Supplementary file 1 [file molecules-26-06774-s001.zip › molecules-1448570-supplementary.pdf]

SUPPLEMENTARY MATERIALS

The participation of 3,3,3-trichloro-1-nitroprop-1-ene in the [3+2] cycloaddition reaction with selected nitrile N-oxides in the light of the experimental and MEDT quantum chemical study

Karolina Zawadzińska [1], Mar Ríos-Gutiérrez [2*], Karolina Kula [1], Przemysław Woliński [1], Barbara Mirosław [3], Tomasz Krawczyk [4], and Radomir Jasiński [1*]

[1] Department of Organic Chemistry and Technology, Cracow University of Technology, Warszawska 24, 31-155 Cracow, Poland.
[2] Department of Organic Chemistry, University of Valencia, Dr. Moliner 50, Burjassot, 46100 Valencia, Spain.
[3] Department of General and Coordination Chemistry and Crystallography, Maria Curie-Sklodowska University in Lublin, Pl. Marii Curie-Sklodowskiej 3, 20-031 Lublin, Poland.
[4] Department of Chemical Organic Technology and Petrochemistry, Silesian University of Technology, Krzywoustego 4, 44-100 Gliwice, Poland.
* Correspondence: rios@utopia.uv.es (M.R.G.); radomir@chemia.pk.edu.pl (R.J.)

INDEX

3	**MATERIALS**
3	**ANALYTICAL TECHNIQUES**
3	**X-RAY CRYSTAL STRUCTURE DETERMINATION**
3	**GENERAL PROCEDURE FOR CYCLOADDITION REACTIONS**
4	**PHYSICAL CHARACTERISTICS OF SYNTHESIZED 2-ISOXAZOLINE**
16	**Table S1** – Crystal X-Ray diffraction data for Δ^2-isoxazoline (**3a**)
17	**Table S2** – Bond lengths (Å) and valence angles (°) for Δ^2-isoxazoline (**3a**)
17	**Table S3** – Geometry of interactions in of crystal Δ^2-isoxazoline (**3a**)
18	**Table S4** – Populations of the most relevant ELF valence basins of NOs **1a,c,d**, in average number of electrons, $\bar{e}$.
18	**Table S5** – B3LYP/6-31G(d) HOMO and LUMO energies used for the calculation of the global CDFT indices of NOs **1a-e**, in eV.
18	**Table S6** – Nucleophilic P_k^- Parr functions at the O1 and C3 atoms of NOs **1a,c,d**.
19	**Table S7** – *ω*B97X-D/6-311G(d,p) electronic energies in gas phase and in tetrahydrofuran (THF), in a.u., of the reagents and stationary points involved in the 32CA reactions of NOs **1a-e** with TNP **2**.
20	**Table S8** – *ω*B97X-D/6-311G(d,p) enthalpies (H, in a.u.), entropies (S, in cal·mol^{-1}K^{-1}), and Gibbs free energies (G, in a.u.), and the relative ones with respect to the separated reagents, computed at 25 ºC, in THF, of the reagents and stationary points involved in the 32CA reaction of NOs **1a-e** with TNP **2**.
21	**Figure S1** – *ω*B97X-D/6-311G(d,p) optimized geometries, in THF, of the regioisomeric TSs involved in the 32CA reaction of NOs **1a,c,d** with TNP **2**.
22	**Figure S2** – *ω*B97X-D/6-311G(d,p) intrinsic reaction coordinate paths, in THF, of the 32CA reactions between NOs **1a,e** and TNP **2**.

23 LITERATURE

MATERIALS

All hydroxamoyl chlorides (**1a-e**) and (*E*)-3,3,3-trichloro-1-nitroprop-1-ene (TNP) **2** were prepared in reactions described earlier in literature [1-3]. Commercially available solvents were used as reaction medium.

ANALYTICAL TECHNIQUES

For reaction testing, a Knauer apparatus equipped with UV-VIS detector, and Lichrospher 100-5 RP18 column (4 x 250) was applied. Methanol – water mixtures (70:30 v/v) were used as eluent at the flow rate 1.0 cm^3 min^{-1}. Melting points were determined on the Boetius apparatus and were not corrected. IR spectra were registered on a FTS Nicolet IS 10 apparatus. ^1H NMR (400 MHz) and ^{13}C NMR (100 MHz) spectra were taken on an Agilent 400 MHz spectrometer using $CDCl_3$ as a solvent. HR-MS spectra were performed on a Waters Xevo G2 QTof instrument using ESI ionization. Spectrum recording was determined for the 200-500nm range by usage of spectrometer UV-5100 BIOSENS.

X-RAY CRYSTAL STRUCTURE DETERMINATION

The diffraction intensities were collected at room temperature with the use of SuperNova X-ray diffractometer with Atlas S2 CCD detector using the mirror-monochromatized Cu$K\alpha$ radiation (λ= 1.54184 Å). The phase problem was solved by direct methods using the SHELXS-97 program and the structure model refined by the full-matrix least-squares method on F^2 using the SHELXL-97 program implemented in Olex2.refine package [4,5].

GENERAL PROCEDURE FOR CYCLOADDITION REACTIONS

Erlenmeyer flask was put in an ice bath. The 10 ml of THF was dosed, then 0,003 mole 3,3,3-trichloro-1-nitroprop-1-ene **2** and 0,0024 mole of proper hydroxamoyl chloride (**1a-e**) was added and stirred for 10 min. Next the 0,0015 mole K_2CO_3 was dosed in small amounts for 30 min. After that time, the ice bath was removed. Change of mixture colour and turbidity was observed. The mixture was left for 24 hours. The mixture was left to solvent evaporation, next the main product was washed off with diethyl ether, solid side product doesn't dissolve in ether. Ether was then evaporated due to usage of vacuum distillation. The obtained semisolid mass was washed by could petroleum eter and crystallised from ethanol. A white crystalline solid was obtained.

PHYSICAL CHARACTERISTICS OF SYNTHESIZED 2-ISOXAZOLINE

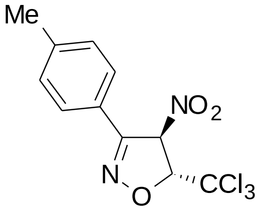

***4,5-trans*-3-(4-methylphenyl)-4-nitro-5-trichloromethyl-Δ²-isoxazoline** (**3a**)

$C_{11}H_9N_2O_3Cl_3$. White crystal needles obtained in 68.85% yield; m.p. 78 – 80°C from ethanol; IR: υ = 1563 and 1366 (NO_2), 1259 (-C-O-N-), 1610 (-C=N-), 805 ($C(Cl_2)$-Cl) cm^{-1}; UV 265 nm; ^1H NMR (400 MHz, $CDCl_3$): δ 7.7 (d, 2H, J=8.61Hz); 7.3 (d, 2H, J=8.61Hz); 6.4 (d, 1H, J=3.91Hz); 5.7 (d, 1H, J=3.91Hz); 2.4 (s, 3H); ^{13}C NMR (100 MHz, $CDCl_3$): δ 151.6; 142.6; 130; 126.9; 94.2; 93.2; 21.5; HR–MS (ESI-): calculated for $C_{11}H_8N_2O_3Cl_3$ $[M-H]^-$ = 320.96005, found 320.9601;

IR

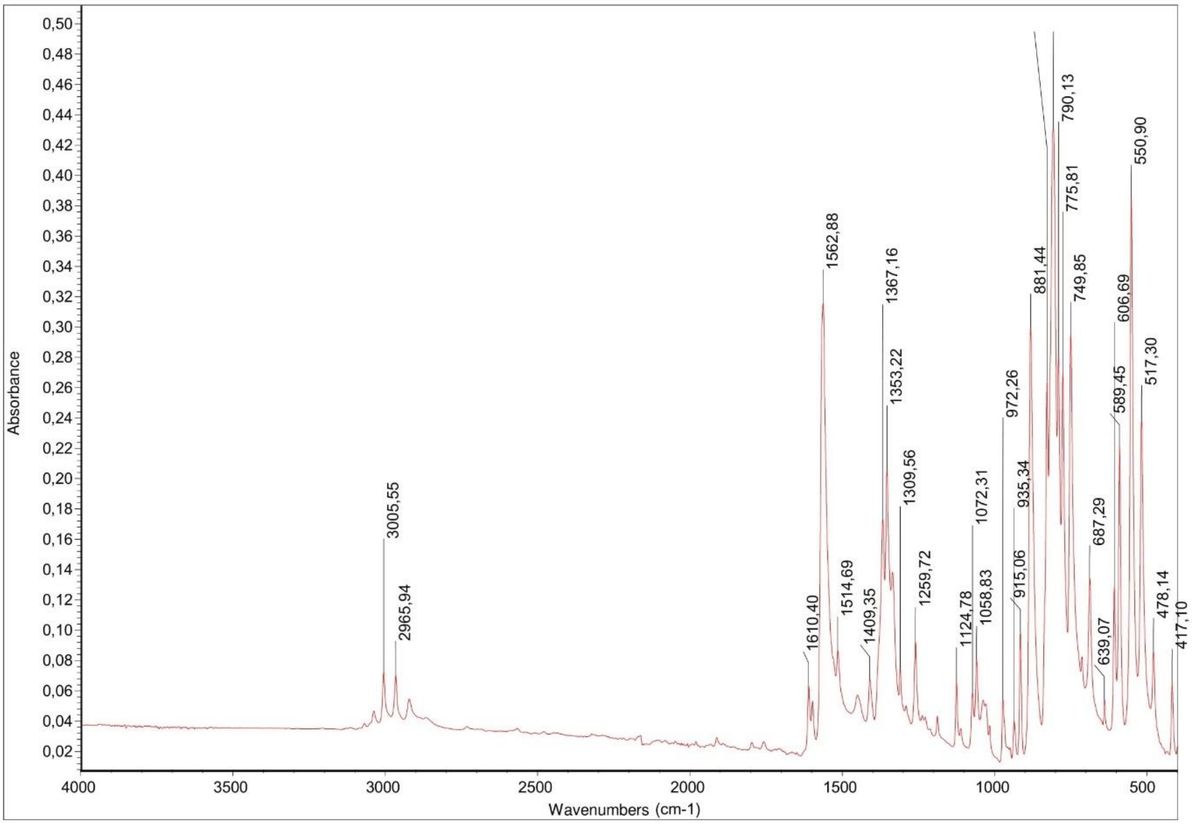

^{1}H NMR

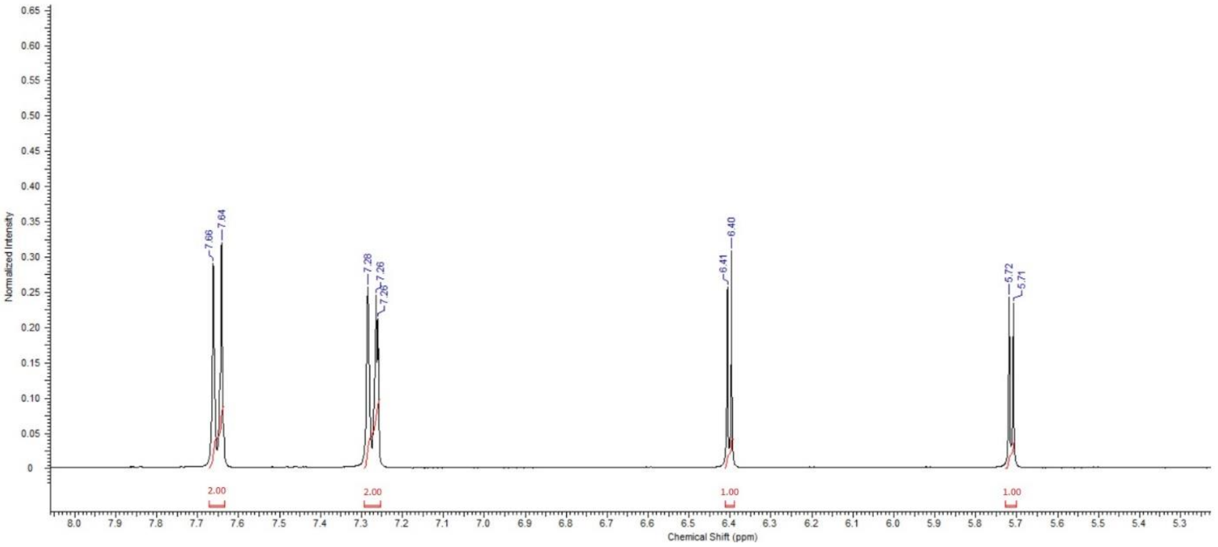

^{13}C NMR

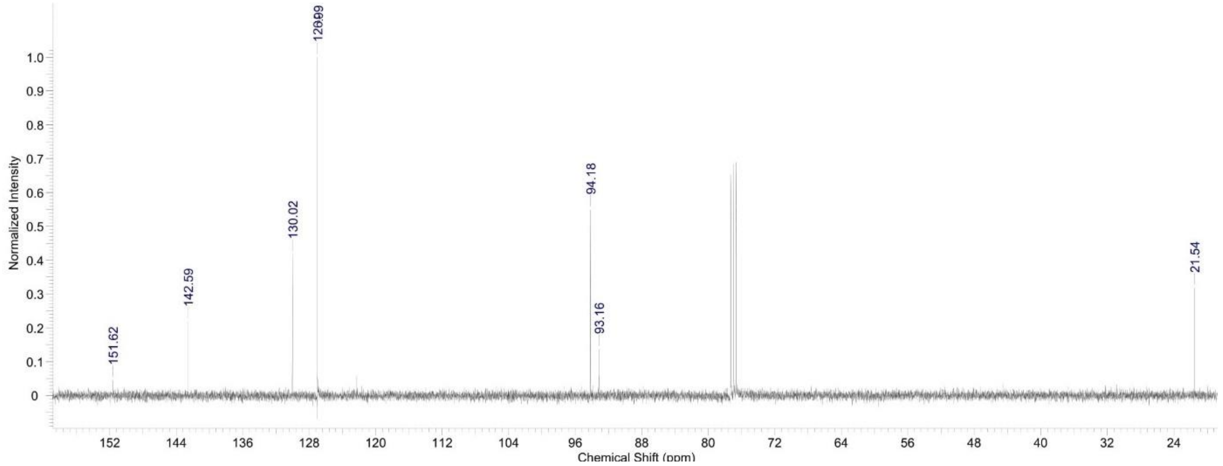

HR-MS

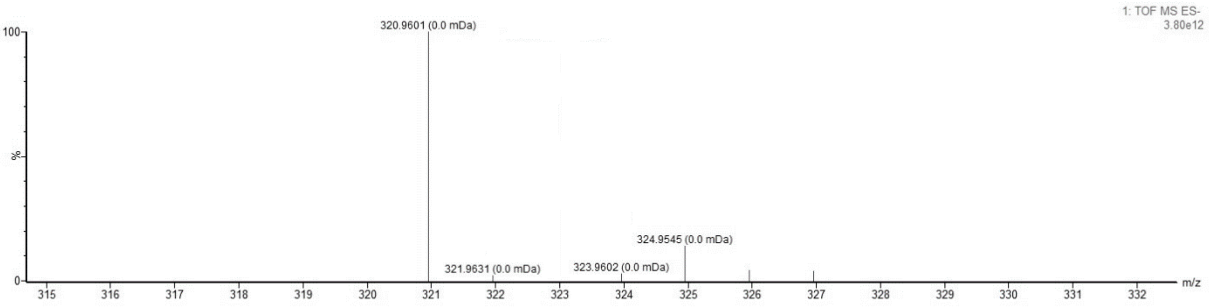

COSY

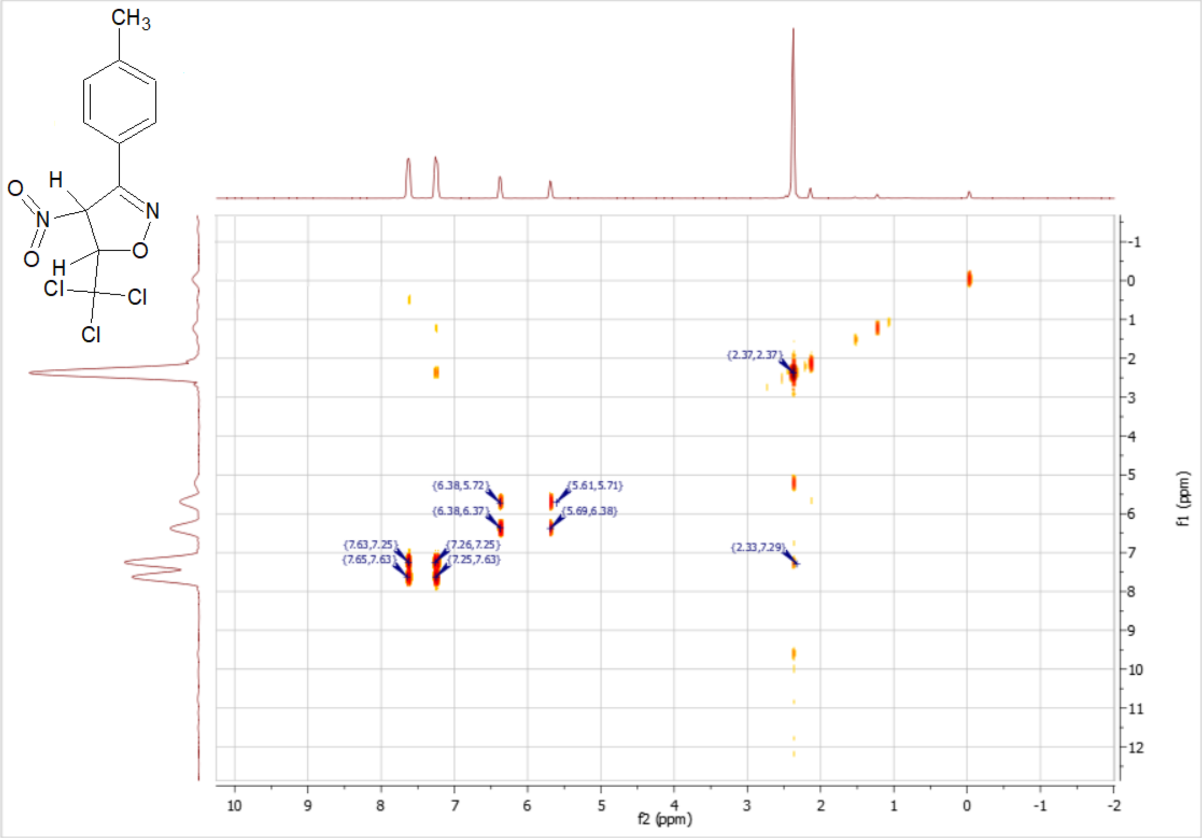

CH3
H
O
N
N
O
O
H
Cl
Cl
Cl
{2.37,2.37}
{6.38,5.72}
{5.61,5.71}
{6.38,6.37}
{5.69,6.38}
{7.63,7.25}
{7.65,7.63}
{7.26,7.25}
{7.25,7.63}
{2.33,7.29}
f2 (ppm)
f1 (ppm)

NOESY

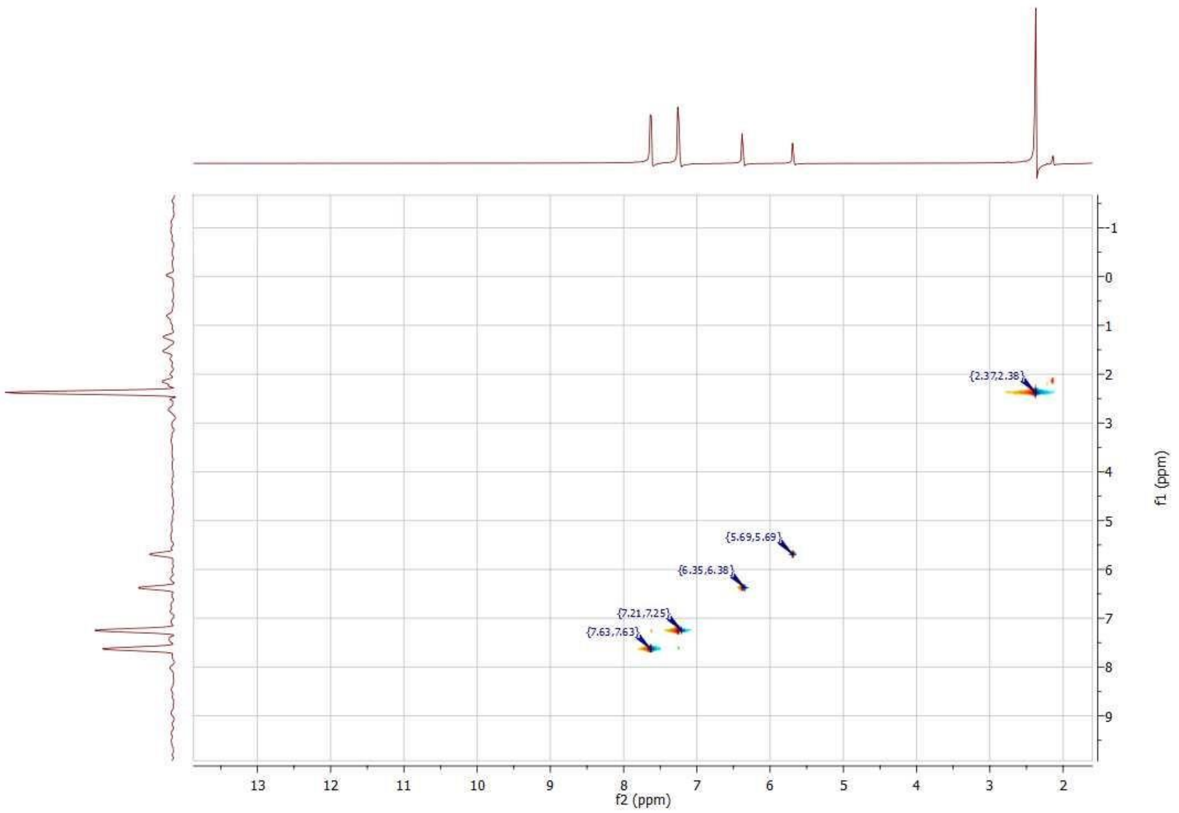
{2.37,2.38}
{5.69,5.69}
{6.35,6.38}
{7.21,7.25}
{7.63,7.63}
f2 (ppm)
f1 (ppm)

HSQC

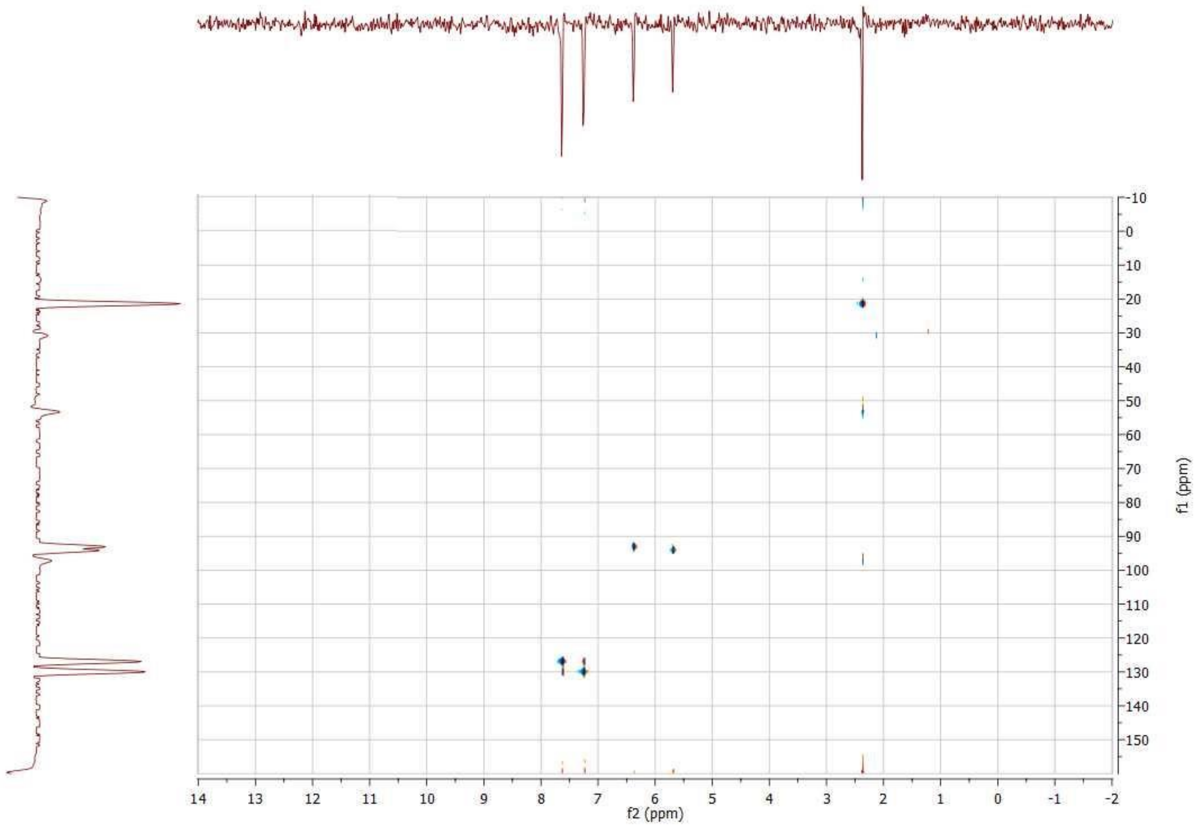
f2 (ppm)
f1 (ppm)

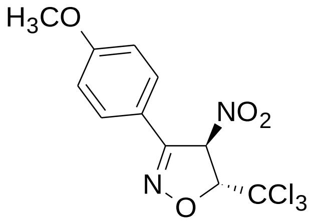

4,5-*trans*-3-(4-methylphenyl)-4-nitro-5-trichloromethyl-Δ²-isoxazoline (**3b**)

$C_{12}H_9N_2O_4Cl_3$. White crystal needles obtained in 43,07 % yield; m.p. 63 – 66°C from ethanol; IR: υ = 1563 and 1349 (NO_2), 1262 (-C-O-N-), 1609 (-C=N-), 815 ($C(Cl_2)$-Cl) cm^{-1}; UV 277 nm; ^1H NMR (400 MHz, $CDCl_3$): δ 7.71 (d, 2H, J=9.00Hz); 6.97 (d, 2H, J=9.00Hz); 6.39 (d, 1H, J=3.91Hz); 5.71 (d, 1H, J=3.91Hz); 3.86 (s, 3H), ^{13}C NMR (100 MHz, $CDCl_3$): δ 160.55; 149.31; 126.91; 112.93; 92.24; 91.46; 53.6; HR–MS (ESI+): calculated for $C_{12}H_9N_2O_4Cl_3$ $[M+H]^+$ = 382.9345, found 382.9343;

IR

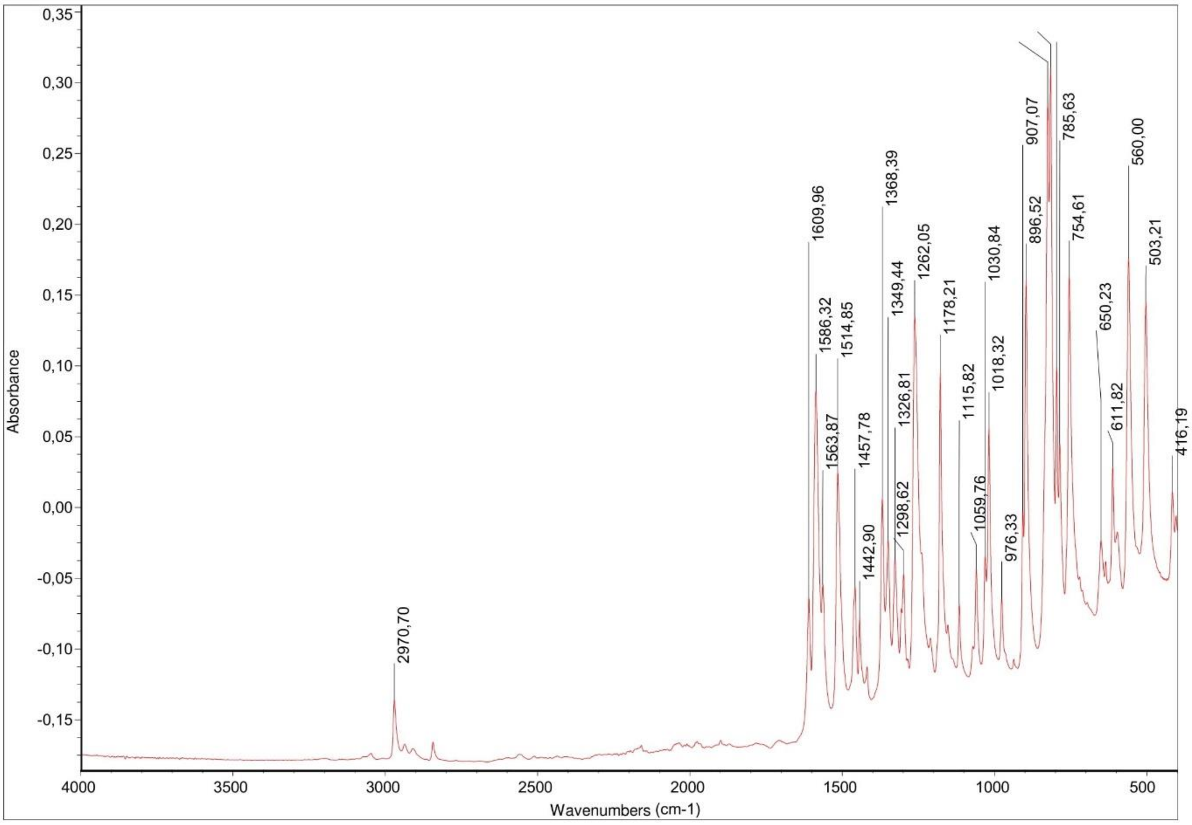

^{1}H NMR

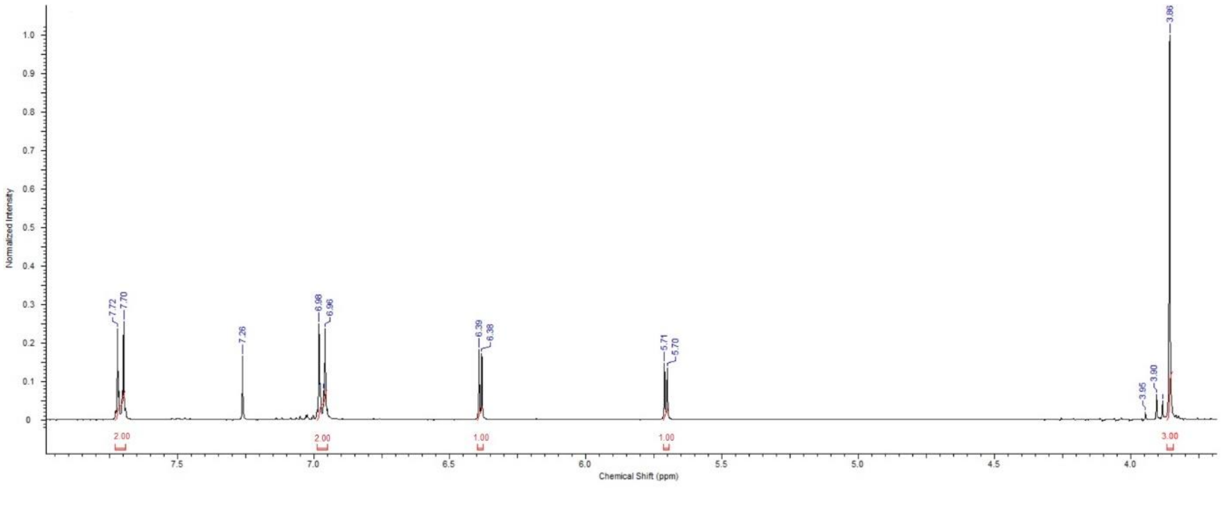

^{13}C NMR

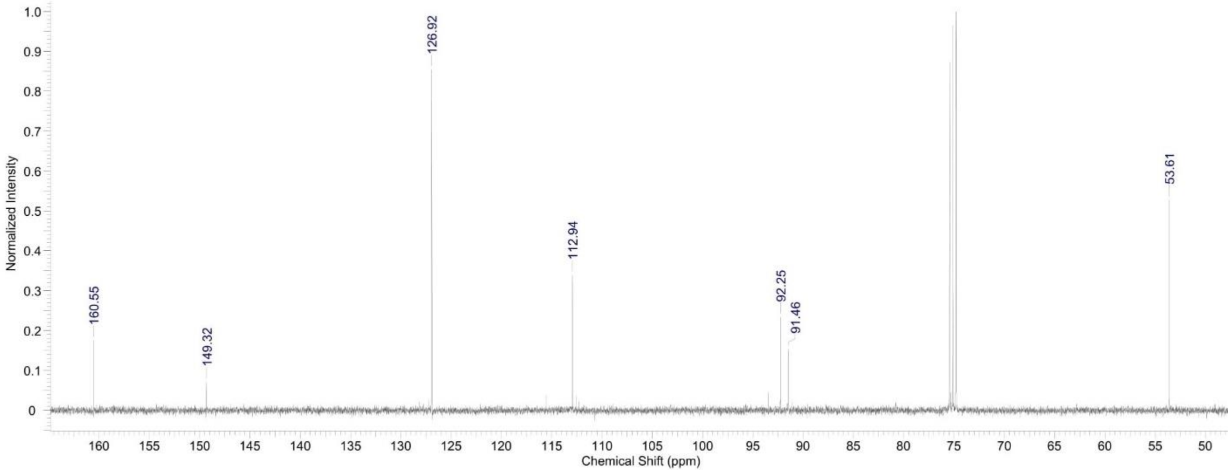

HR-MS

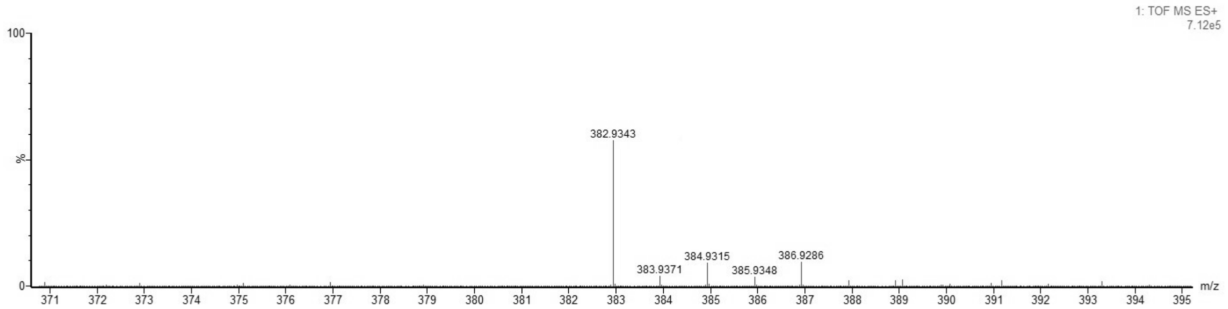

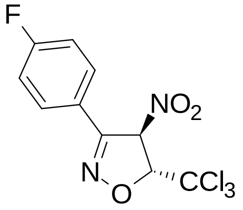

***4,5-trans*-3-(4-fluorophenyl)-4-nitro-5-trichloromethyl-Δ²-isoxazoline** (**3c**)

$C_{10}H_6N_2O_3Cl_3F$. White crystal needles obtained in 70% yield; m.p. 104 – 106°C from ethanol; IR: υ = 1563 and 1365 (NO_2), 1256 (-C-O-N-), 1600 (-C=N-), 815 ($C(Cl_2)$-Cl) cm^{-1}; UV 258 nm; ^1H NMR (500 MHz, $CDCl_3$): δ 7.75-7.78 (dd, 2H, J=5.09Hz); 7.16 (t, 2H, J=8.61Hz); 6.37 (d, 1H, J=3.91Hz); 5.74 (d, 1H, J=3.91Hz); ^{13}C NMR (100 MHz, $CDCl_3$): δ 165.98 163.45 (d, J=253Hz); 150.71; 129.36; 129.27; 116.81; 94.38; 92.95; ^{19}F NMR (376 MHz): δ -106.24, (m, 1F); HR–MS (ESI+): calculated for $C_{10}H_6N_2O_3Cl_3F$ $[M+H]^+$ = 370.9145, found 370.9145;

IR

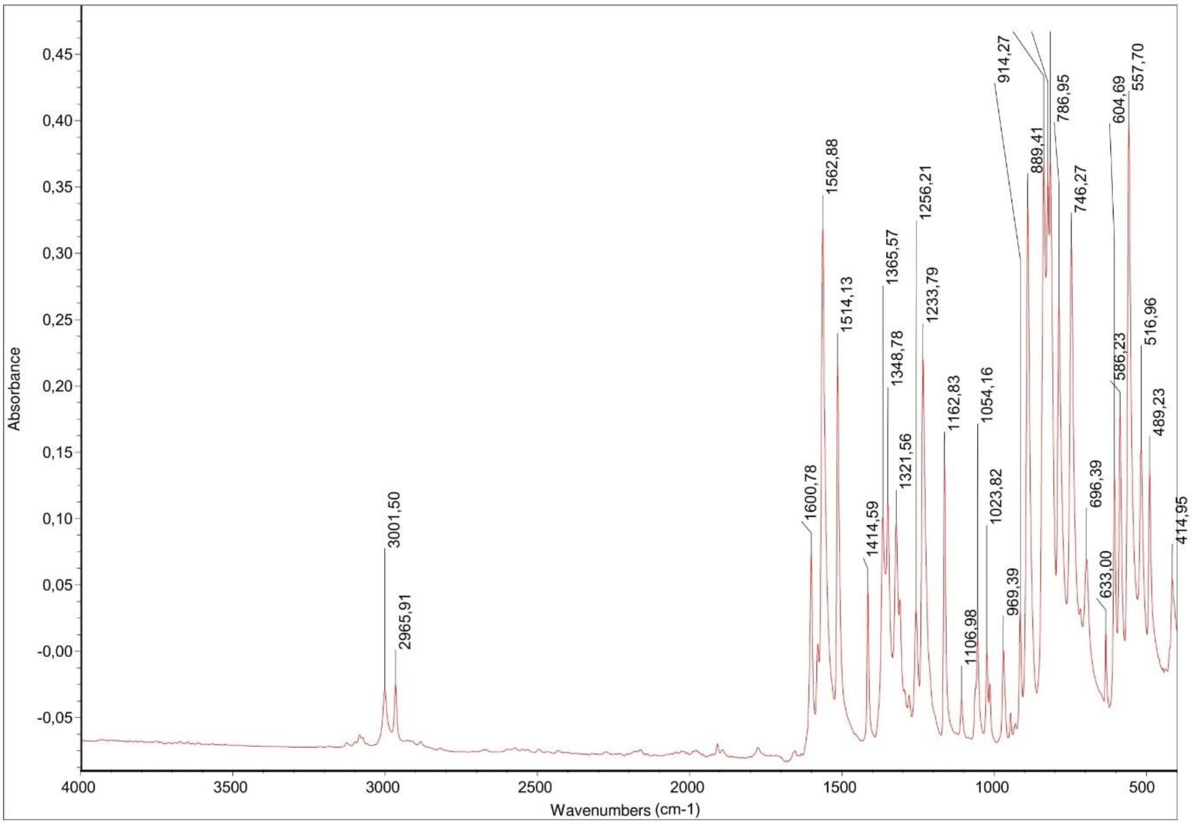

^{1}H NMR

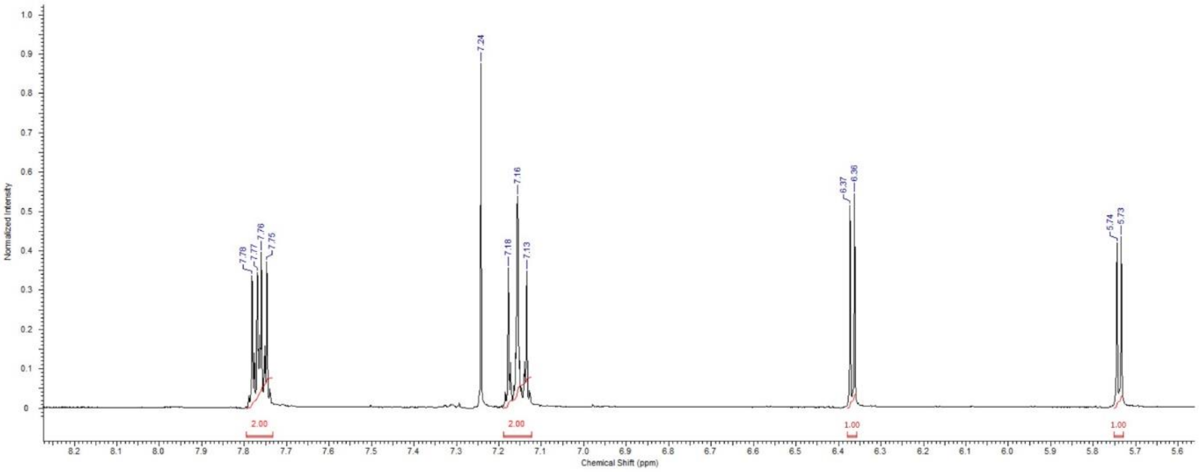

^{13}C NMR

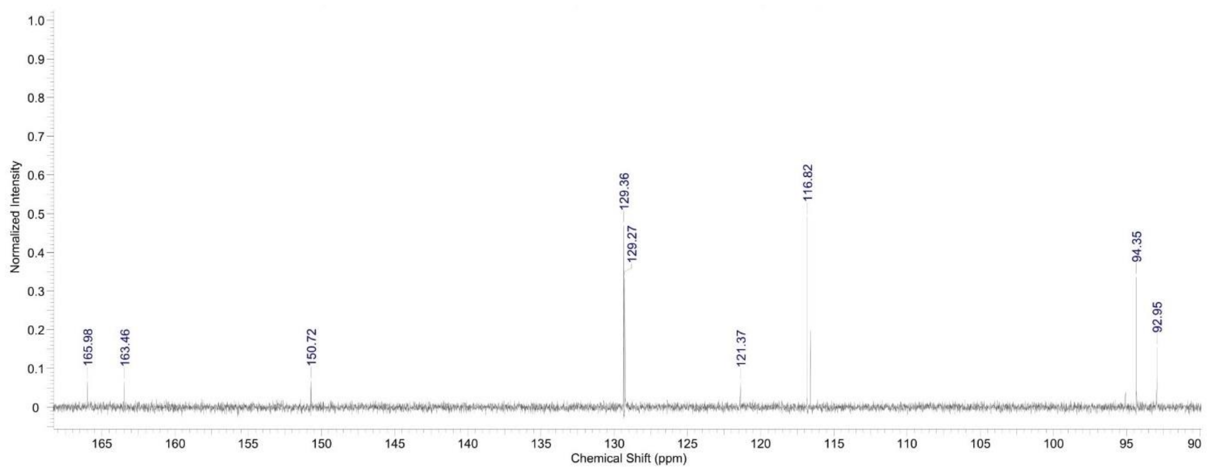

HR-MS

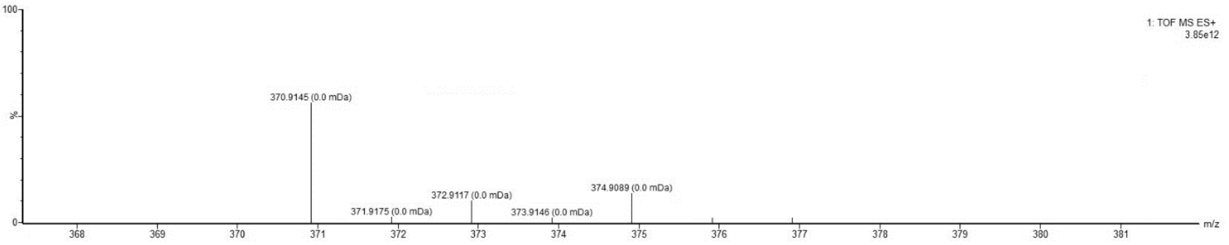

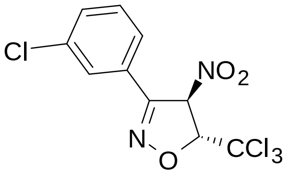

4,5-trans**-3-(3-chlorophenyl)-4-nitro-5-trichloromethyl-Δ²-isoxazoline** (**3d**)

$C_{10}H_6N_2O_3Cl_4$. White crystal needles obtained in 40 % yield; m.p. 58 – 61°C from ethanol; IR: υ = 1557 and 1354 (NO_2), 1252 (-C-O-N-), 1591 (-C=N-), 819 ($C(Cl_2)$-Cl) cm^{-1}; UV 258 nm; ^1H NMR (400 MHz, $CDCl_3$): δ 7.79 (t, 1H, J=1.96Hz); 7.62-7.64 (dd, 1H, J=1.17Hz); 7.49-7.51 (dd, 1H, J=1.17Hz); 7.42 (d ,1H); 6.38 (d, 1H, J=3.91Hz); 5.77 (d, 1H, J=3.91Hz); ^{13}C NMR (100 MHz, $CDCl_3$): δ 150.67; 131.91; 130.55; 127.03; 126.87; 94.45; 92.51; HR–MS (ESI+): calculated for $C_{10}H_6N_2O_3Cl_4$ $[M+H]^+$ = 386.8850, found 386.8850;

IR

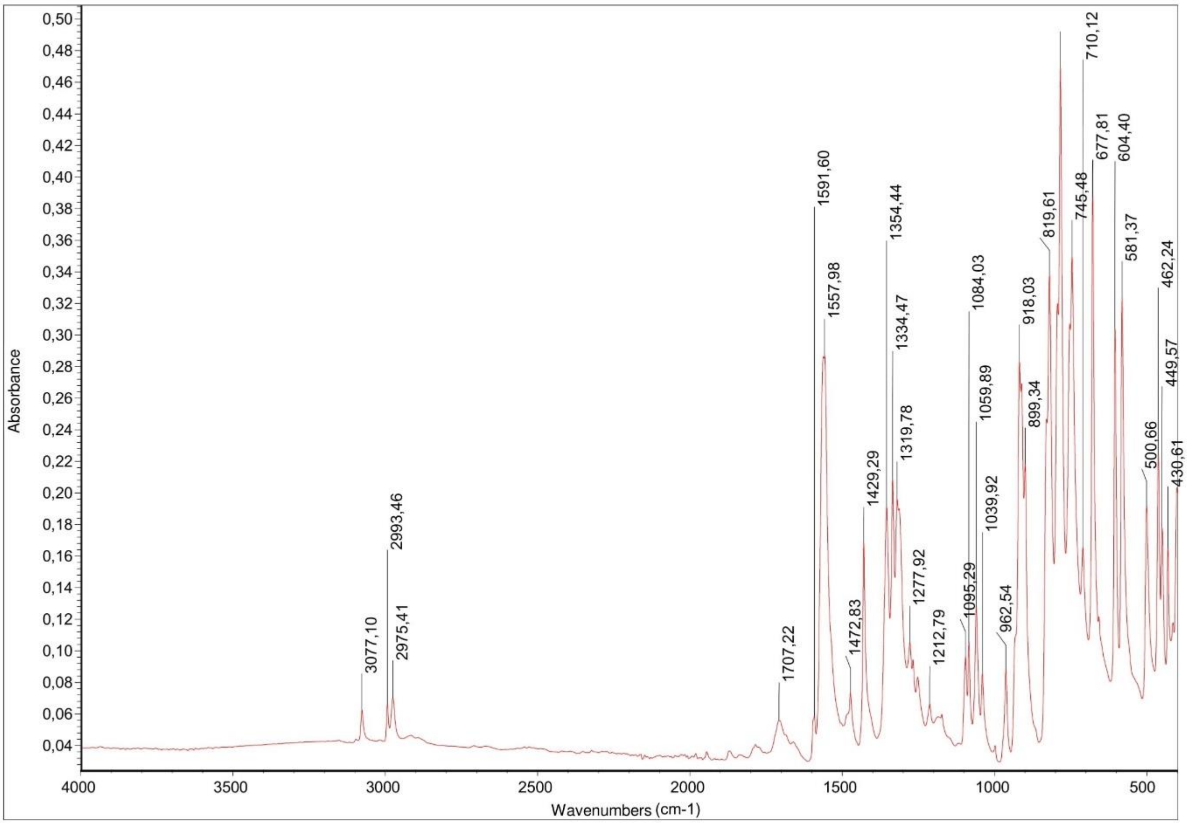

[1]<u>H NMR</u>

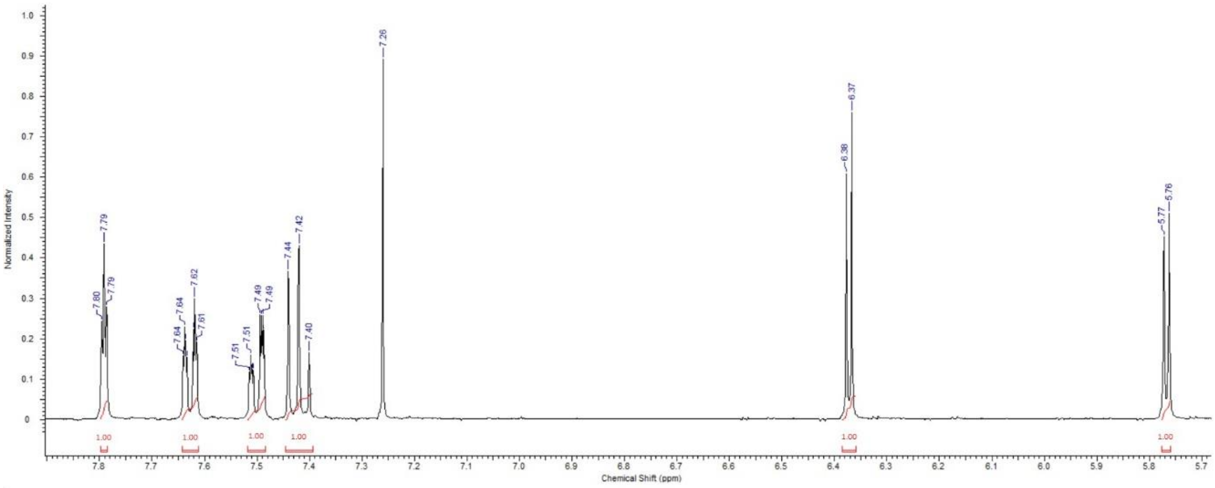

[13]<u>C NMR</u>

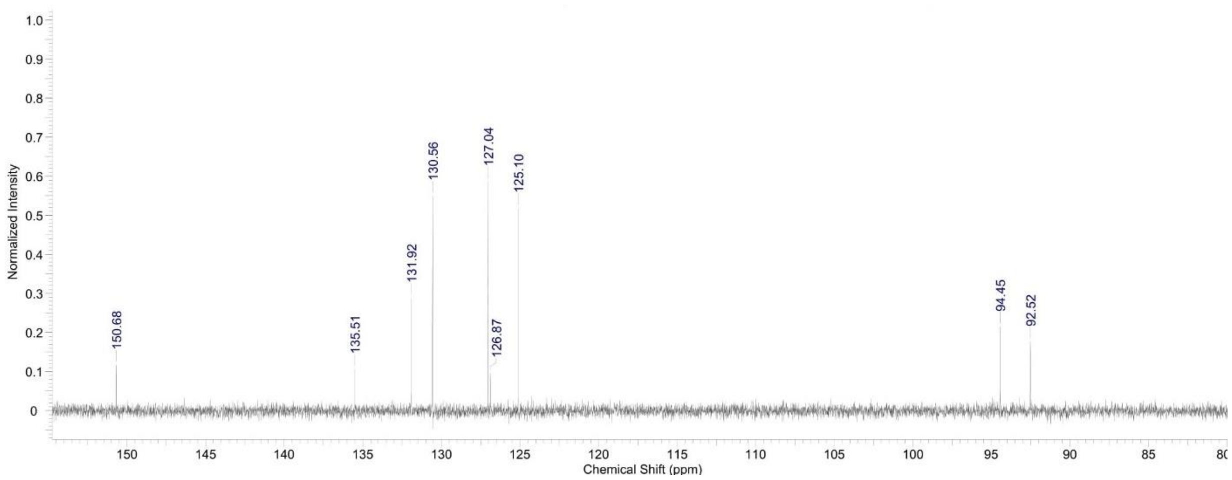

HR-MS

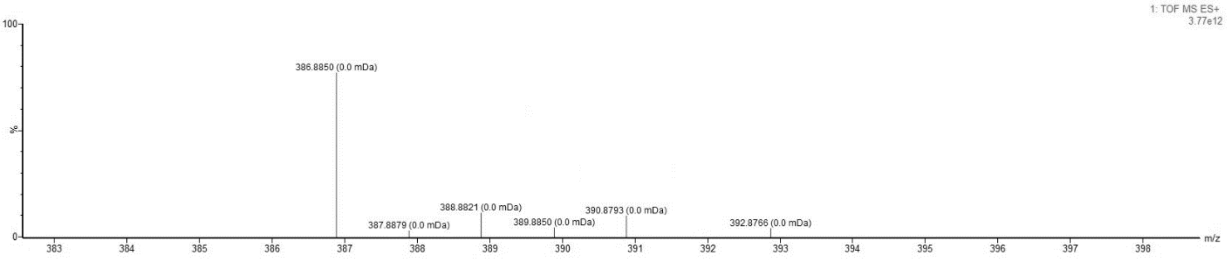

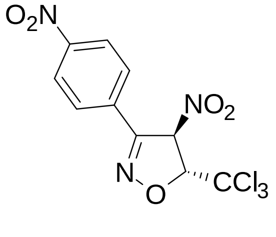

***4,5-trans*-3-(4-nitrophenyl)-4-nitro-5-trichloromethyl-Δ²-isoxazoline** (**3e**)

$C_{10}H_6N_3O_5Cl_3$. White crystal needles obtained in 40% yield; m.p. 115 – 120°C from ethanol; IR: υ = 1560 and 1341 (NO_2), 1259 (-C-O-N-), 1607 (-C=N-), 817 ($C(Cl_2)$-Cl) cm^{-1}; UV 283 nm; ^1H NMR (400 MHz, $CDCl_3$): δ 8.32-8.34 (m, 2H, J=1.96Hz); 7.97-7.99 (m, 2H, J=1.96Hz); 6.44 (d,1H, J=3.91Hz); 5.86 (d, 1H, J=3.91Hz), ^{13}C NMR (100 MHz, $CDCl_3$): δ 150.3; 131.05; 128.4; 128.1; 124.5; 94.79; 92.02; HR–MS (ESI+): calculated for $C_{10}H_6N_3O_5Cl_3$ $[M+H]^+$ = 397.9090, found 397.9090;

IR

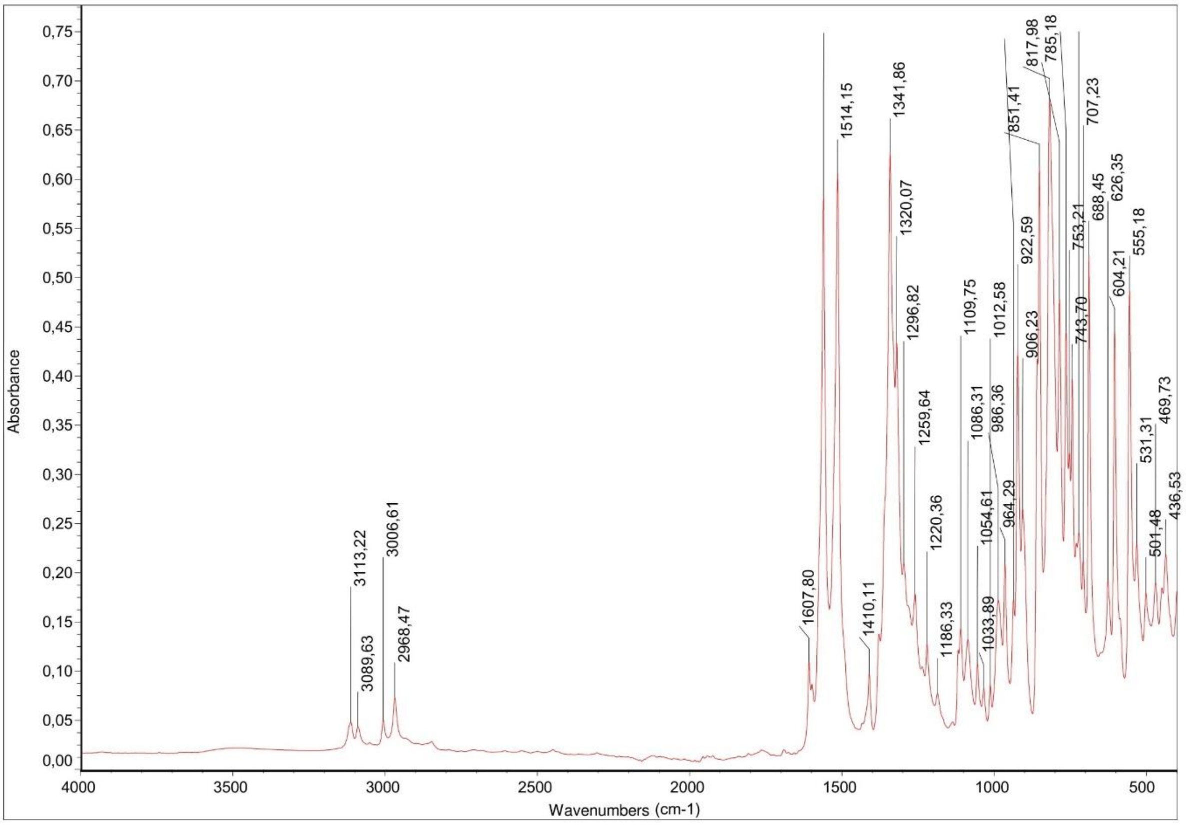

^{1}<u>H</u> <u>NMR</u>

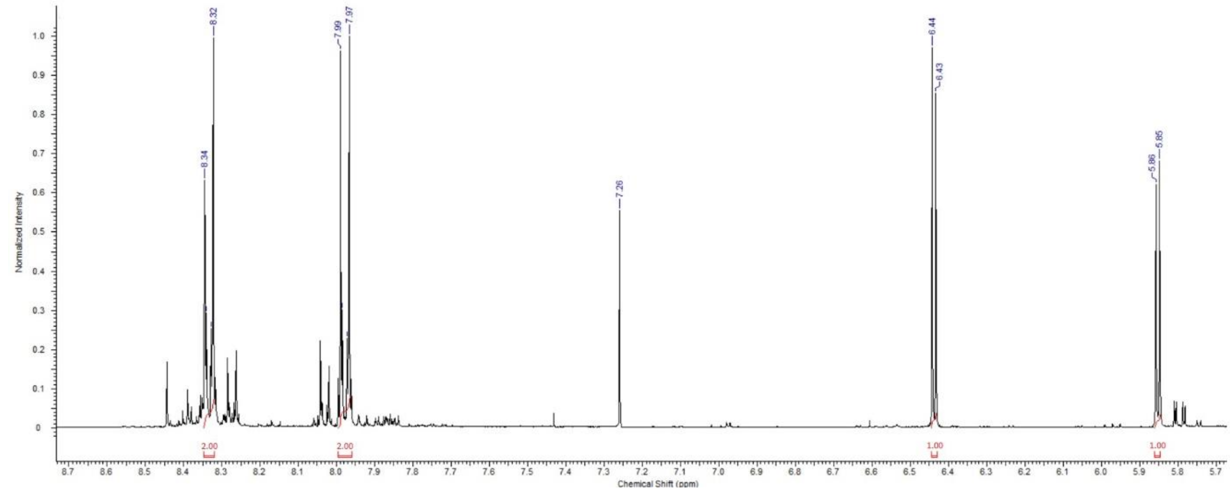

^{13}<u>C</u> <u>NMR</u>

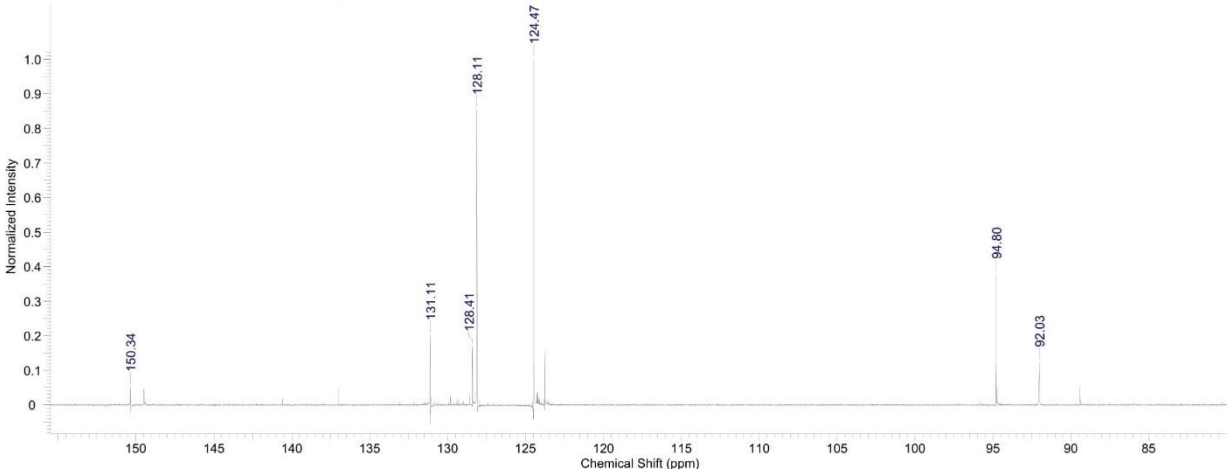

HR-MS

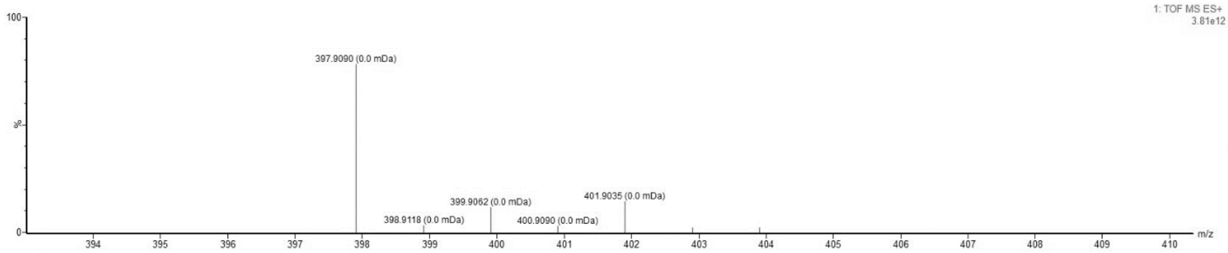

Table S1. Crystal X-Ray diffraction data for Δ[2]-isoxazoline (**3a**)

Empirical formula	$C_{11}H_9Cl_3N_2O_3$
Formula weight	323.55
Temperature/K	294.13(12)
Crystal system	triclinic
Space group	*P*-1
a/Å	5.9745(3)
b/Å	9.3795(4)
c/Å	12.1947(5)
α/°	95.047(4)
β/°	94.403(4)
γ/°	90.605(3)
Volume/Å^3	678.60(5)
Z	2

ρ_{calc}g/cm^3	1.583
μ/mm^{-1}	6.181
F(000)	328.0
Crystal size/mm^3	0.3 × 0.25 × 0.08
Radiation	CuKα (λ = 1.54184)
2Θ range for data collection/°	7.3 to 152.196
Index ranges	$-7 \le h \le 7$, $-11 \le k \le 11$, $-15 \le l \le 15$
Reflections collected	9918
Independent reflections	2778 [R_{int} = 0.0685, R_{sigma} = 0.0466]
Data/parameters	2778/173
Goodness-of-fit on F^2	1.112
Final R indexes [I>=2σ (I)]	R_1 = 0.0509, wR_2 = 0.1342
Final R indexes [all data]	R_1 = 0.0625, wR_2 = 0.1709
Largest diff. peak/hole / e Å^{-3}	0.39/-0.45

Table S2. Bond lengths (Å) and valence angles (°) for Δ^2-isoxazoline (**3a**).

Bond		Angle	
C1-C2	1.533(3)	N1-O1-C2	109.8(2)
C1-C3	1.503(4)	C3-N1-O1	110.3(2)
C2-C4	1.522(4)	C3-C1-C2	101.5(2)
N1-C3	1.278(4)	O1-C2-C1	104.7(2)
N2-C1	1.513(3)	N1-C3-C1	112.9(2)
O1-N1	1.412(3)		
O1-C2	1.437(3)		
O2-N2	1.209(3)		
O3-N2	1.217(3)		
Cl1-C4	1.777(3)		
Cl2-C4	1.757(3)		
Cl3-C4	1.770(3)		

Table S3. Geometry of interactions in of crystal Δ^2-isoxazoline (**3a**).

	D–H	H…A	D…A	<DHA	Symmetry code
C1-H1…O3	0.98	2.38	3.224(3)	144	1-x,1-y,1-z
C2-H2…O3	0.98	2.47	3.57(3)	151	-x,1-y,1-z
C7-H7…O2	0.93	2.64	3.322(4)	131	-1+x,y,z
C6-H6…O2	0.93	2.89	3.444(4)	119	-1+x,y,z
C6-H6…O3	0.93	2.75	3.675(4)	175	-1-x,-1-y,-1-z
C1-H1…O3	0.98	2.38	3.224(3)	144	-1-x,-1-y,-1-z
C11-H11C…N1	0.96	2.88	3.415(4)	116	-x,-1-y,-z
C7-H7…Cl2	0.93	3.02	3.790(4)	142	-1+x,-1+y,z
O2…O3	-	-	3.094(4)	-	-x,-1-y,-z
O3…N2	-	-	2.996(3)	-	-x,-1-y,-1-z
O3…O3	-	-	2.985(3)	-	-x,-1-y,-1-z
Cl1…Cl1	-	-	3.610(1)	-	-1-x,-y,-1-z

Table S4. Populations of the most relevant ELF valence basins of NOs **1a,c,d**, in average number of electrons, $\bar{e}$.

	**1c** (4-F)	**1d** (3-Cl)	**1a** (4-Me)
V(O1,N2)	1.69	1.69	1.69
V(N2)			
V(N2,C3)	3.01	3.03	2.88
V'(N2,C3)	3.01	2.97	3.12
V(C3,C3')	2.47	2.47	2.48
V(O1)	5.66	2.84	2.97
V'(O1)	0.01	2.82	2.70

Table S5. B3LYP/6-31G(d) HOMO and LUMO energies used for the calculation of the global CDFT indices of NOs **1a-e**, in eV.

	HOMO	LUMO

**1a** (4-Me)	-6.1715	-1.2033
**1b** (4-MeO)	-5.8788	-0.9709
**1c** (4-F)	-6.3579	-1.3347
**1d** (3-Cl)	-6.6020	-1.6444
**1e** (4-NO_2)	-7.0513	-3.0240
TNP **2**	-8.4557	-3.2308

Table S6. Nucleophilic P_k^- Parr functions at the O1 and C3 atoms of NOs **1a,c,d**.

	O1	C3
**1a**	0.46	-0.01
**1c**	0.48	0.01
**1d**	0.49	0.03

Table S7. ωB97X-D/6-311G(d,p) electronic energies in gas phase and in tetrahydrofuran (THF), in a.u., of the reagents and stationary points involved in the 32CA reactions of NOs **1a-e** with TNP **2**.

	Gas phase	THF
TNP **2**	-1701.175692	-1701.181209
**1a** (4-Me)	-438.905608	-438.911770
**TS-o-a**	-2140.069477	-2140.078354
**TS-m-a**	-2140.066633	-2140.075326
**3a**	-2140.159582	-2140.167339
**4a**	-2140.156646	-2140.168020
**1b** (4-MeO)	-514.108307	-514.116031
**TS-o-b**	-2215.273093	-2215.283620
**TS-m-b**	-2215.270182	-2215.280290
**3b**	-2215.362462	-2215.371673
**4b**	-2215.359532	-2215.372431
**1c** (4-F)	-498.826633	-498.832821
**TS-o-c**	-2199.990093	-2199.998886

**TS-m-c**	-2199.987234	-2199.996009
**3c**	-2200.080840	-2200.088552
**4c**	-2200.077945	-2200.089331
**1d** (3-Cl)	-859.193278	-859.199210
**TS-o-d**	-2560.355678	-2560.364305
**TS-m-d**	-2560.353197	-2560.361861
**3d**	-2560.447366	-2560.455112
**4d**	-2560.444574	-2560.455945
**1e** (4-NO2)	-604.075969	-604.084758
**TS-o-e**	-2305.236783	-2305.248301
**TS-m-e**	-2305.234645	-2305.246355
**3e**	-2305.329894	-2305.340458
**4e**	-2305.327068	-2305.341280

Table S8. ωB97X-D/6-311G(d,p) enthalpies (H, in a.u.), entropies (S, in cal·mol^{-1}K^{-1}), and Gibbs free energies (G, in a.u.), and the relative ones with respect to the separated reagents, computed at 25 ºC in THF, of the reagents and stationary points involved in the 32CA reaction of NOs **1a-e** with TNP **2**.

	H	ΔH	S	ΔS	G	ΔG
TNP **2**	-1701.115302		98.977		-1701.162329	
**1a** (4-Me)	-438.770319		95.140		-438.815523	
**TS-o-a**	-2139.870667	9.4	148.370	-45.7	-2139.941162	23.0
**TS-m-a**	-2139.867642	11.3	146.941	-47.2	-2139.937459	25.3
**3a**	-2139.955391	-43.8	142.961	-51.2	-2140.023317	-28.5
**4a**	-2139.956252	-44.3	141.959	-52.2	-2140.023701	-28.8
**1b** (4-MeO)	-513.968497		97.479		-514.014813	
**TS-o-b**	-2215.069809	8.8	149.427	-47.0	-2215.140806	22.8
**TS-m-b**	-2215.066484	10.9	148.680	-47.8	-2215.137127	25.1
**3b**	-2215.153608	-43.8	144.303	-52.2	-2215.222172	-28.3
**4b**	-2215.154666	-44.5	143.490	-53.0	-2215.222842	-28.7
**1c** (4-F)	-498.728080		89.353		-498.770535	
**TS-o-c**	-2199.827946	9.7	142.690	-45.6	-2199.895743	23.3

**TS-m-c**	-2199.825038	11.5	141.861	-46.5	-2199.892441	25.4
**3c**	-2199.913252	-43.8	136.082	-52.2	-2199.977909	-28.3
**4c**	-2199.914310	-44.5	135.676	-52.7	-2199.978774	-28.8
**1d** (3-Cl)	-859.095438			92.2		-859.1
**TS-o-d**	-2560.194223	10.4	144.178	-47.0	-2560.262727	24.4
**TS-m-d**	-2560.191869	11.8	144.450	-46.7	-2560.260501	25.8
**3d**	-2560.280777	-43.9	139.291	-51.9	-2560.346958	-28.5
**4d**	-2560.281889	-44.6	138.253	-52.9	-2560.347578	-28.9
**1e** (4-NO2)	-603.967323			99.8		-604.0
**TS-o-e**	-2305.064409	11.4	151.407	-47.4	-2305.136347	25.6
**TS-m-e**	-2305.062585	12.6	151.148	-47.7	-2305.134401	26.8
**3e**	-2305.152414	-43.8	146.975	-51.8	-2305.222246	-28.3
**4e**	-2305.153457	-44.4	146.127	-52.7	-2305.222887	-28.7

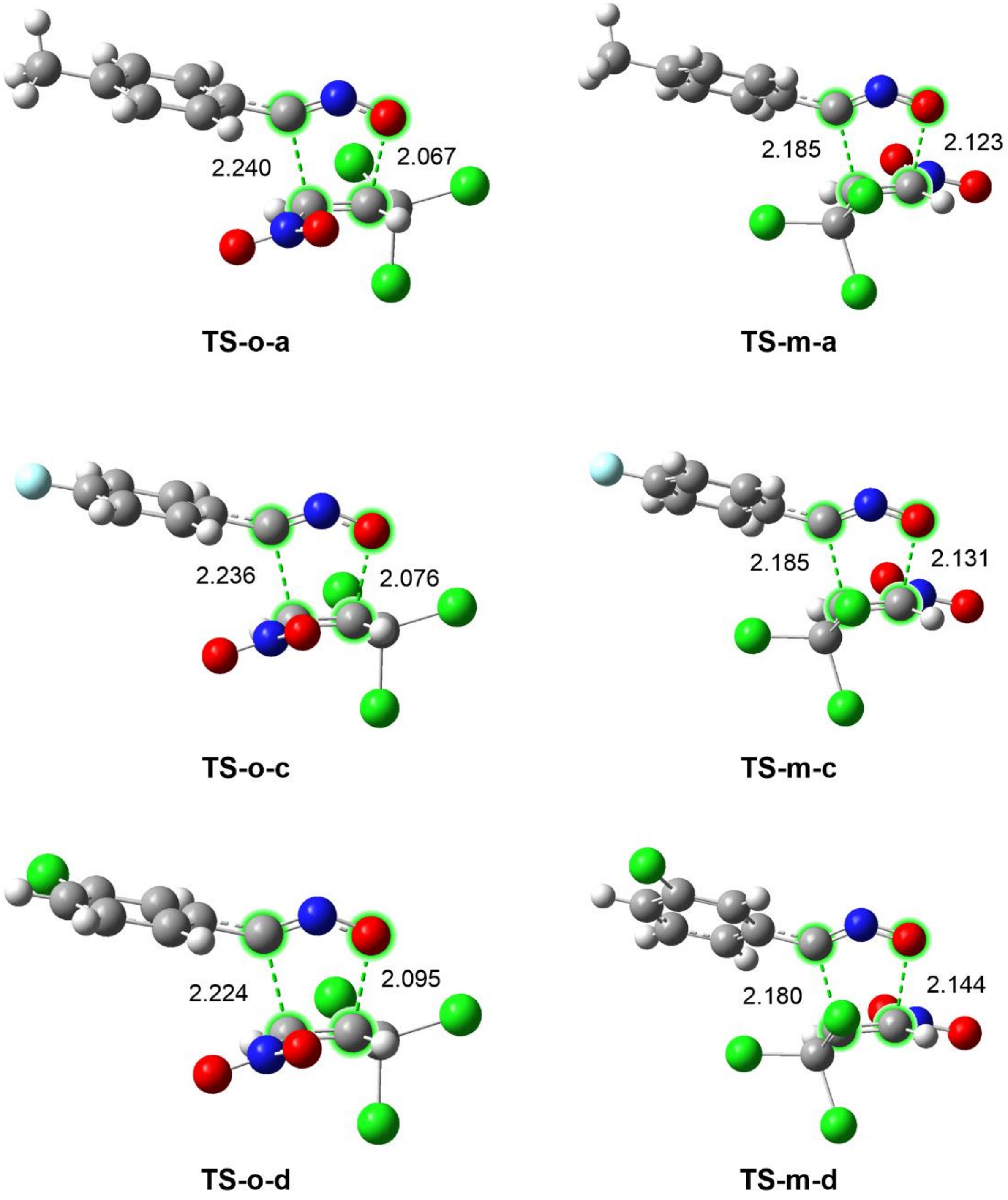

Figure S1. *ω*B97X-D/6-311G(d,p) optimized geometries in THF of the regioisomeric TSs involved in the 32CA reaction of NOs **1a,c,d** with TNP **2**.

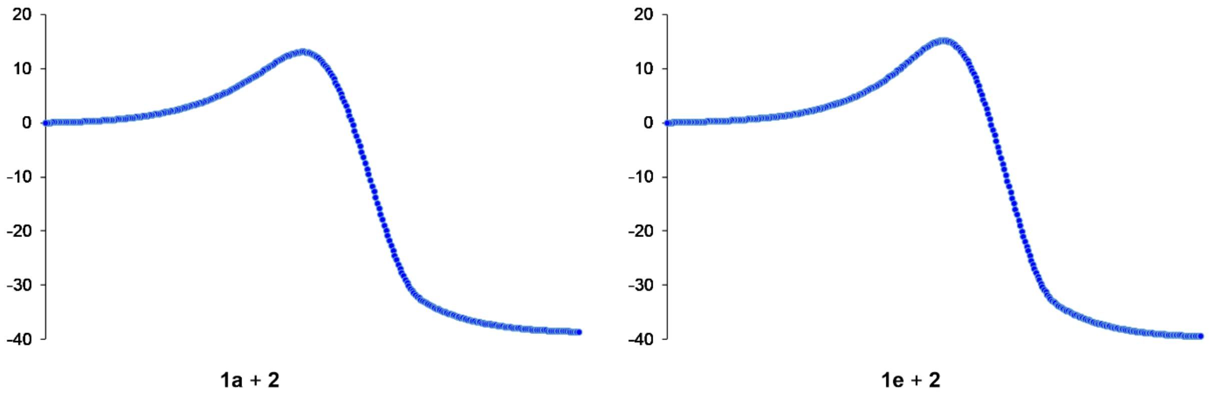

Figure S2. ωB97X-D/6-311G(d,p) intrinsic reaction coordinate paths, in THF, of the 32CA reactions between NOs **1a,e** and TNP **2**. Relative energies with respect to the first structure of the reaction paths are given in kcal·mol^{-1}.

LITERATURE

1. Llopis, Q.; Guillamot, G.; Phansavath, P.; Ratovelomanana-Vidal V. Enantioselective Synthesis of α-Acetal-β′-Amino Ketone Derivatives by Rhodium-Catalyzed Asymmetric Hydrogenation. *Org.Lett.* **2017** *19*, 6428–6431.
2. Liu, K.-Ch.; Shelton, B.R.; Howe, R.K. A particularly convenient preparation of benzohydroximinoyl chlorides (nitrile oxide precursors). *J. Org. Chem.* **1980**, *45*, 3916–3918.
3. Perekalin, W.; Lipina, E.S.; Berestovitskaya, V.M.; Efremov, D.A. *Nitroalkenes: Conjugated Nitrocompounds,* 1st ed.; Wiley: New York, USA, 1994.
4. Sheldrick, G.M. A short history of SHELX. *Acta Crystallogr. Sect. A* **2008**, *64*, 112–122.
5. Dolomanov, O. V.; Bourhis, L.J.; Gildea, R.J.; Howard, J.A.K.; Puschmann, H. OLEX2: A complete structure solution, refinement and analysis program. J. Appl. Crystallogr. 2009, 42, 339–341.
